# Approximation-based adaptive fixed-time tracking control for uncertain high-order nonlinear systems subject to time-varying parameters and unknown input nonlinearity

**DOI:** 10.1038/s41598-025-90830-6

**Published:** 2025-03-26

**Authors:** Xiyu Zhang, Zhi Yang, Youjun Zhou, Xiongfeng Deng

**Affiliations:** 1 School of Mathematics and Computer Science, Guangxi Science and Technology Normal University, Laibin, 546199 China; 2 School of Mechanical and Electrical Engineering, Guang’an Institute of Technology, Guang’an, 638550 China; 3 Key Laboratory of Electric Drive and Control of Anhui Higher Education Institutes, Anhui Polytechnic University, Wuhu, 241000 China

**Keywords:** High-order nonlinear systems, Fixed-time tracking control, Time-varying parameter, Input nonlinearity, Applied mathematics, Information technology

## Abstract

In this paper, the fixed-time tracking control (FTTC) problem is discussed for a type of uncertain high-order nonlinear systems. Compared with the existing works, the studied system is affected by time-varying parameters and unknown input nonlinearity. By applying neural network (NN) approximation method together with the adaptive control method, the fixed-time control theory, the backstepping control method, and the Nussbaum gain function (NGF) technique, an adaptive NN-based FTTC scheme is presented to achieve fixed time tracking. Especially, the NGF is utilized to handle the unknown control gain caused by unknown input nonlinearity. Furthermore, some adaptive control laws are formulated to estimate unknown parameters. Under the influence of different input nonlinearity, it can be inferred that the designed control strategy guarantees that the tracking error converges to a small neighborhood of zero within a fixed time, while also maintaining the boundedness of all signals of the closed-loop system. Finally, three simulation cases are presented to validate the availability of the theoretical results.

## Introduction

In recent decades, the control problems of various nonlinear systems have received widespread attention, and many meaningful achievements have also been made. For instance, Shahvali et al.^1,2^ proposed adaptive NN dynamic surface tracking control strategy and output-feedback event-triggered control law for the control problems of stochastic nonlinear systems and uncertain nonlinear systems. Deng et al., Pan et al. and Cheng et al.^3–5^ discussed the control problems of uncertain nonlinear systems with the strict-feedback form, in which the studied systems were further affected by unknown control coefficients, unknown actuator fault and time-varying delays. Wang et al. and Cai et al.^6,7^ addressed the non-strict feedback nonlinear systems, where the control issues of the predefined time tracking control and the finite-time tracking control were solved, respectively. Moreover, Zhang et al. and Jia^8,9^ investigated the control problems of pure-feedback nonlinear systems, and the designed control schemes can ensure that the given system achieved zero tracking error within a predefined time and no-overshooting control, respectively. Furthermore, the tracking control problems of non-affine nonlinear systems with actuator constraints, input quantization and DoS attack, and dead-zone inputs were further studied^10–13^. Additionally, Bali et al.^14,15^ developed the control schemes with hybrid neural network for uncertain switched nonlinear systems to abtain the desired tracking control. Some control problems of other nonlinear systems, such as the stochastic interconnected nonlinear systems and the fully actuated systems^16,17^, have also received attention. From the above results, it can be seen that the research on the control problems of nonlinear systems is still a hot topic. However, it is worth noting that these nonlinear systems mentioned above have one thing in common, that is, their order is one. When the order of the system is no longer one, how to design the control strategy will be a topic worth studying.

For nonlinear systems with orders no longer equal to one, known as HONSs, many existing control methods cannot be directly applied^10–17^. To address the control challenges associated with such HONSs, several effective control strategies have been suggested. For instance, Zhou et al. and Liu et al.^18,19^ studied HONSs with unknown control gains and unknown powers, in which the fuzzy-based approximate control methods were proposed to achieve tracking control. Moreover, the prescribed performance asymptotic tracking control problems of HONSs with odd rational power were explored^20,21^, in which the considered systems can achieved asymptotic tracking under the prescribed performance. Furthermore, the finite-time optimal stabilization problem for a type of switched HONSs was investigated^22^, wherein the orders of the system can take various odd rational values under the designated switched signal. Meanwhile, the global finite-time stability can be achieved by using the nested saturation homogeneous control strategy. Additionally, the prescribed-time control issue for a class of HONSs with actuator faults was developed^23^, while Wang^24^ studied the high-order nonlinear switched systems from the perspective of adaptive fixed time tracking control.

From the above mentioned results and references therein^18–24^, it can be seen that many works have been done on the control problem of HONSs, there are still some shortcomings. For example, the nonlinear dynamics considered were required to satisfy the Lipschitz condition^19,21^. In the works of Gao et al. and Wang et al.^23,24^, the unknown control gain caused by unknown actuator faults, input saturation and dead-zone fault was assumed to be a bounded constant, which is obviously somewhat conservative. Moreover, although the control problem of HONSs with unknown control gains discussed by Zhou et al., Lv et al. and Li et al.^18,20,22^, they did not conduct in-depth research on the problem of system input nonlinearity, and only Lv et al.^20^ considered the existence of input quantization. However, due to the actual system may be affected by various factors, the system model may have unknown time-varying parameters and may also experience different input nonlinearities. Fully considering these constraints has great practical significance for the control of HONSs.

On the other hand, it is clearly meaningless to demand that the control problem of the actual system be implemented in an infinite time. However, the implementation of many control problems mentioned above is based on the infinite time^16–21^, which is obviously not very reasonable. Therefore, some scholars have explored the control problems of the system from the perspectives of the finite-time control, the prescribed-time control, and the fixed-time control. Bali et al. and Xi et al.^25,26^ studied the finite-time tracking control issue, where the designed controllers ensured that the tracking error converges to a small neighborhood of the origin within a finite time. Moreover, the prescribed-time stability problems of nonlinear systems with different constraint conditions were addressed^27–29^, in which the given systems can achieve pre-specified tracking performances within a prescribed time. Furthermore, for the fixed-time tracking problem of nonlinear systems, adaptive FTTC strategies to guarantee achieve fixed-time output tracking were proposed^30–32^. Additionally, the finite-time control, the prescribed-time control, and the fixed-time control problems of practical systems were addressed^33–35^, and good control effects were achieved under the designed control strategies.

Upon reviewing the aforementioned works, it becomes evident that there is a scarcity of discourse surrounding the fixed-time control problem for HONSs, which further motivates our investigation into this matter. Therefore, the FTTC problem for a class of uncertain HONSs is studied in this paper. The system under consideration is affected by unknown time-varying parameters and unknown input nonlinearity. Based on the adaptive control method, the approximation-based method, the fixed-time control theory, the backstepping control technique and the NGF method, an adaptive NN-based FTTC scheme is proposed to achieve the fixed time tracking. The main highlights of this paper are as follows.

i. Different from some discussed systems^18–23^, the HONSs considered in this paper subjects to odd integer powers and uncertain nonlinear dynamics, and the unknown time-varying parameters and the unknown input nonlinearity are further considered. Obviously, the proposed model exhibits greater complexity and generality.
ii. Unlike the actor-critic reinforcement learning method^36,37^, the radial basis function (RBF) NN-based approximation method is applied to approximate the uncertain dynamics of the system and unknown nonlinearities generated during the analysis process, which effectively simplifies the difficulty of designing control laws. At the same time, the unknown time-varying parameters of the system and the derivative of virtual control laws generated during the application of backstepping control process are effectively handled through the adaptive control method.
iii. To solve the issue of unknown control gain caused by unknown input nonlinearity, the NGF technique is introduced in this paper. Then, an adaptive NN-based FTTC strategy is developed by combining fixed-time control theory and NN control method to achieve the fixed time tracking. Compared to the infinite time tracking control problem^16–19^, this paper solves the FTTC problem. Obviously, the problem discussed in this paper has more practical significance.
iv. The application of the proposed control method can effectively ensure that the tracking error converges to a small neighborhood of zero within a fixed time under different unknown input nonlinearities, and all signals in the closed-loop system can remain bounded.

The rest of this paper is arranged as follows. In Section “Problem formulation and preliminaries”, the system description and preliminaries are given, including a brief introduction to the RBFNN. In Section “Control law design and stability analysis”, the design of adaptive NN-based FTTC law is presented, and the stability analysis is also provided. Finally, the simulation analysis and main conclusions are included in Sections “Simulation analysis” and “Conclusion”, respectively.

## Problem formulation and preliminaries

### System description

Consider the following uncertain HONSs with time-varying parameters

1 $$\begin{aligned} \dot{x}_{i} & = x_{i + 1}^{{\rho_{i} }} + \vartheta_{i}^{T} (t)\phi_{i} (\overline{x}_{i} ) + f_{i} (\overline{x}_{i} ),\;i = 1, \ldots ,n - 1 \\ \dot{x}_{n} & = \left( {{\mathcal{D}}(u)} \right)^{{\rho_{n} }} + \vartheta_{n}^{T} (t)\phi_{n} (\overline{x}_{n} ) + f_{n} (\overline{x}_{n} ) \\ y & = x_{1} \\ \end{aligned}$$

where $\overline{x}_{i} = [x_{1} , \ldots ,x_{i} ]^{T} \in R^{i}$, $i = 1, \ldots ,n$, and $y \in R$ are the system’s states and output, $\vartheta_{i} (t) \in R^{j}$, $i = 1, \ldots ,n$, $j = 1, \ldots ,n_{0}$, represent unknown time-varying parameter vectors, $\phi_{i} (\overline{x}_{i} ) \in R^{j}$, $i = 1, \ldots ,n$, $j = 1, \ldots ,n_{0}$, represent known smooth nonlinear function vectors, $f_{i} (\overline{x}_{i} ) \in R$, $i = 1, \ldots ,n$, represent uncertain nonlinear dynamics, $\rho_{i} \ge 1$, $i = 1, \ldots ,n$, represent positive odd integers, ${\mathcal{D}}(u) \in R$ represents the system input subjects to unknown nonlinearity. Here, the unknown nonlinearity model is defined as

2 $${\mathcal{D}}(u) = \kappa_{1} (t)u(t) + \kappa_{2} (t)$$

where $u(t)$ represents the actual control signal, $0 < \kappa_{1,\min } < \left| {\kappa_{1} (t)} \right| \le \kappa_{1,\max }$ and $0 \le \left| {\kappa_{2} (t)} \right| \le \kappa_{2,\max }$ with $\kappa_{1,\min } > 0$, $\kappa_{1,\max } > 0$ and $\kappa_{2,\max } > 0$ are unknown constants.

#### Remark 1

The input nonlinearity model described by Eq. (2) is more general. Some input nonlinearities, such as the actuator fault^3,23^, the dead-zone fault^7,12,13,30^, and the input quantization^20,32^, can all be unified in the form of (2).

The objective of this paper is to develop an effective NN-based adaptive FTTC strategy for system (1), ensuring that the system’s output tracks the desired trajectory $y_{d}$ within a fixed time, while also guaranteeing the boundedness of all signals in the closed-loop system.

#### Assumption 1

The desired trajectory $y_{d}$ and its first time derivative $\dot{y}_{d}$ are continuous, known and bounded.

#### Assumption 2

Time-varying parameter vectors $\vartheta_{i} (t)$ and nonlinear function vectors $\phi_{i} (\overline{x}_{i} )$, $i = 1, \ldots ,n$, are bounded.

### RBFNN

An RBFNN $w^{T} \psi (X)$ is utilized to approximate any unknown nonlinear function $Y(X)$^8,24^, which is described by

3 $$Y(X) = w^{T} \psi (X)$$

where $X \in \Omega_{X} \subset R^{n}$ and $w = [w_{1} , \ldots ,w_{l} ]^{T} \in R^{l}$ represent input vector and weight vector, $\psi (X) = [\psi_{1} (X), \ldots ,\psi_{l} (X)]^{T}$ denotes the basis function vector with $\psi_{i} (X)$ being selected as the Gaussian functions , $i = 1, \ldots ,l$, where $\text{B}_{i} = [\text{B}_{i1} , \ldots ,\text{B}_{in} ]^{T}$ represents the center vector and a_*i*_ stands for the width.

Then, for a unknown nonlinear function ${\mathcal{G}}(X)$ over the compact set $\Omega_{X} \subset R^{n}$, there is an RBFNN $(w^{*} )^{T} \psi (X)$ such that for $\varepsilon (X) > 0$, one has

4 $${\mathcal{G}}(X) = (w^{*} )^{T} \psi (X) + \varepsilon (X)$$

where $\varepsilon (X)$ represents the approximation error and satisfies $\left| {\varepsilon (X)} \right| \le \Xi$ with $\Xi > 0$ being a unknown constant, and $w^{*}$ represents the ideal weight vector, which is defined as

5 $$w^{*} : = \arg \mathop {\min }\limits_{{w \in R^{l} }} \left\{ {\mathop {\sup }\limits_{{X \in \Omega_{X} }} \left| {{\mathcal{G}}(X) - w^{T} \psi (X)} \right|} \right\}$$

### Definitions and lemmas

#### Definition 1

^3^A smooth function ${\mathcal{N}}(\chi )$, if satisfies the following properties.

6 $$\left\{ \begin{gathered} \mathop {\lim }\limits_{s \to \infty } \sup \frac{1}{s}\int_{0}^{s} {{\mathcal{N}}(\chi )d\chi } = + \infty \hfill \\ \mathop {\lim }\limits_{s \to \infty } \inf \frac{1}{s}\int_{0}^{s} {{\mathcal{N}}(\chi )d\chi } = - \infty \hfill \\ \end{gathered} \right.$$

then it is called as Nussbaum-type function. Here, the Nussbaum-type function ${\mathcal{N}}(\chi ) = \exp (\chi^{2} )\cos (\pi \chi /2)$ is considered in this paper.

#### Lemma 1

^3^*Let* $$\chi (t)$$ *on* $$[0,t_{f} )$$ *be smooth function*, $$V(t)$$ *be a positive definite function, and* $${\mathcal{N}}(\chi )$$ *be Nussbaum-type function. If the following inequality holds*

7 $$V(t) \le a_{0} + e^{{( - a_{1} t)}} \int_{0}^{t} {e^{{(a_{1} \tau )}} \left( {G(\tau ){\mathcal{N}}(\chi ) + 1} \right)\dot{\chi }d\tau }$$

*where* $$a_{0}$$ *and* $$a_{1}$$ *are positive constants*, $$G(\tau )$$ *is non-zero but bounded time-varying parameter*, *then* $$V(t)$$, $$\chi (t)$$ *and* $$\int_{0}^{t} {e^{{(a_{1} \tau )}} \left( {G(\tau ){\mathcal{N}}(\chi ){ + }1} \right)\dot{\chi }d\tau }$$ *are bounded on* $$[0,t_{f} )$$.

#### Lemma 2

^24^*A nonlinear system is described by* $\dot{x} = {\mathcal{T}}(x)$, *where* $x \in R^{n}$ *is the state and* ${\mathcal{T}}(x)$ *satisfies* ${\mathcal{T}}(0) = 0$. *If there is a positive definite function* $V(x)$ *satisfies*

8 $$\dot{V}(x) \le - {\mathfrak{m}}_{1} V^{q} (x) - {\mathfrak{m}}_{2} V^{p} (x) + {\mathfrak{m}}_{0}$$

*where* ${\mathfrak{m}}_{0}$, ${\mathfrak{m}}_{1}$ *and* ${\mathfrak{m}}_{2}$ *are positive constants*, $0 < q < 1$ *and* $p > 1$, *then the system (8) is practically fixed-time stable and* $V(x)$ *is able to stabilize to the minor region around zero, that is*

9 $$V(x)_{{t \to T_{s} }} \le \min \left\{ {\left( {\frac{{{\mathfrak{m}}_{0} }}{{{\mathfrak{m}}_{1} (1 - {\mathfrak{y}})}}} \right)^{{{1 \mathord{\left/ {\vphantom {1 q}} \right. \kern-0pt} q}}} ,\left( {\frac{{{\mathfrak{m}}_{0} }}{{{\mathfrak{m}}_{2} (1 - {\mathfrak{y}})}}} \right)^{{{1 \mathord{\left/ {\vphantom {1 p}} \right. \kern-0pt} p}}} } \right\}$$

10 $$T_{s} \le T_{\max } : = \frac{1}{{{\mathfrak{m}}_{1} {\mathfrak{y}}(1 - q)}} + \frac{1}{{{\mathfrak{m}}_{2} {\mathfrak{y}}(p - 1)}}$$

*where* $$0 < {\mathfrak{y}} < 1$$.

#### Lemma 3

^18^*For all* ${\mathfrak{g}}_{1} \in R$ *and* ${\mathfrak{g}}_{2} \in R$, *there is an odd integer* $\rho \ge 1$ *such that*

11 $$\left| {{\mathfrak{g}}_{1}^{\rho } - {\mathfrak{g}}_{2}^{\rho } } \right| \le \rho \left| {{\mathfrak{g}}_{1} - {\mathfrak{g}}_{2} } \right|({\mathfrak{g}}_{1}^{\rho - 1} + {\mathfrak{g}}_{2}^{\rho - 1} )$$

#### Lemma 4

^38^*Consider a separable function* ${\mathcal{W}}(x) = ({\mathfrak{u}}_{1} + {\mathfrak{u}}_{2} )^{\rho }$, *where* ${\mathfrak{u}}_{1} \in R$, ${\mathfrak{u}}_{2} \in R$ *and* $\rho$ *is a positive odd integer. It holds that* ${\mathcal{W}}(x) = \hbar ({\mathfrak{u}}_{1} ,{\mathfrak{u}}_{2} )({\mathfrak{u}}_{1} )^{\rho } + \ell ({\mathfrak{u}}_{1} ,{\mathfrak{u}}_{2} )({\mathfrak{u}}_{2} )^{\rho }$, *where* $\hbar ({\mathfrak{u}}_{1} ,{\mathfrak{u}}_{2} ) \in \left[ {\underline {\hbar } ,\overline{\hbar }} \right]$ *with* $\underline {\hbar } = 1 - {\mathfrak{c}}_{0}$ *and* $\overline{\hbar } = 1 + {\mathfrak{c}}_{0}$, *where* ${\mathfrak{c}}_{0} = \sum\nolimits_{k = 1}^{\rho } {({{\rho !} \mathord{\left/ {\vphantom {{\rho !} {k!(\rho - k)!}}} \right. \kern-0pt} {k!(\rho - k)!}})} (\rho - {k \mathord{\left/ {\vphantom {k \rho }} \right. \kern-0pt} \rho })\varpi^{{{{(\rho } \mathord{\left/ {\vphantom {{(\rho } {\rho - k}}} \right. \kern-0pt} {\rho - k}})}}$ *is an arbitrary constant and its value is within* $(0,1)$ *for an appropriately small constant* $\varpi$, $\left| {\ell ({\mathfrak{u}}_{1} ,{\mathfrak{u}}_{2} )} \right| \le \overline{\ell }({\mathfrak{c}}_{0} ) = \sum\nolimits_{k = 1}^{\rho } {({{\rho !} \mathord{\left/ {\vphantom {{\rho !} {k!(\rho - k)!}}} \right. \kern-0pt} {k!(\rho - k)!}})} ({k \mathord{\left/ {\vphantom {k \rho }} \right. \kern-0pt} \rho })\varpi^{{({\rho \mathord{\left/ {\vphantom {\rho k}} \right. \kern-0pt} k})}}$ *with* $\overline{\ell }({\mathfrak{c}}_{0} )$ *being a positive constant*.

#### Lemma 5

^16^*For* $h_{1} \in R$ *and* $h_{2} \in R$, *and any positive constants* $b_{1}$, $b_{2}$ *and* $b_{3}$, *there is*

12 $$\left| {h_{1} } \right|^{{b_{1} }} \left| {h_{2} } \right|^{{b_{1} }} \le \frac{{b_{1} }}{{b_{1} + b_{2} }}b_{3} \left| {h_{1} } \right|^{{b_{1} + b_{2} }} + \frac{{b_{2} }}{{b_{1} + b_{2} }}b_{3}^{{ - \frac{{b_{1} }}{{b_{2} }}}} \left| {h_{2} } \right|^{{b_{1} + b_{2} }}$$

#### Lemma 6

^21^*For any positive constants* $\sigma$ *and* $\eta$, *there is*

13 $$0 \le \left| \sigma \right| - \frac{{\sigma^{2} }}{{\sqrt {\sigma^{2} + \eta^{2} } }} \le \eta$$

#### Lemma 7

^30^*For* ${\mathfrak{d}}_{i} \ge 0$, $i = 1, \ldots ,n$, $0 < p < 1$ *and *$q > 1$, *there is*

14 $$\left( {\sum\limits_{i = 1}^{n} {{\mathfrak{d}}_{i} } } \right)^{p} \le \sum\limits_{i = 1}^{n} {{\mathfrak{d}}_{i}^{p} } ,\;n^{1 - q} \left( {\sum\limits_{i = 1}^{n} {{\mathfrak{d}}_{i} } } \right)^{q} \le \sum\limits_{i = 1}^{n} {{\mathfrak{d}}_{i}^{q} }$$

## Control law design and stability analysis

### Adaptive NN FTTC law design

Define the following error transformation as

15 $$z_{i} = x_{i} - \upsilon_{i}$$

where $\upsilon_{1} = y_{d}$, and $z_{1}$ represents the tracking error, $\upsilon_{i}$, $i = 2, \ldots ,n$, represent virtual control laws that need to be designed. For the convenience of analysis, $\vartheta_{i} (t)$, $\phi_{i} (\overline{x}_{i} )$, and $f_{i} (\overline{x}_{i} )$, $i = 1, \ldots ,n$, are abbreviated as $\vartheta_{i}$, $\phi_{i}$, and $f_{i}$ without causing confusion.

**Step 1**: Noting the subsystem $\dot{x}_{1} = x_{2}^{{\rho_{1} }} + \vartheta_{1}^{T} \phi_{1} + f_{1}$ and $z_{1} = x_{1} - y_{d}$, then the derivative of $z_{1}$ as

16 $$\dot{z}_{1} = (x_{2}^{{\rho_{1} }} - \upsilon_{2}^{{\rho_{1} }} ) + \upsilon_{2}^{{\rho_{1} }} + \vartheta_{1}^{T} \phi_{1} + f_{1} - \dot{y}_{d}$$

Let $V_{11} = {{z_{1}^{2} } \mathord{\left/ {\vphantom {{z_{1}^{2} } 2}} \right. \kern-0pt} 2}$, the derivative of $V_{11}$ is

17 $$\dot{V}_{11} = z_{1} (x_{2}^{{\rho_{1} }} - \upsilon_{2}^{{\rho_{1} }} ) + z_{1} \upsilon_{2}^{{\rho_{1} }} + z_{1} \left( {\vartheta_{1}^{T} \phi_{1} + f_{1} - \dot{y}_{d} } \right)$$

For the unknown nonlinear function $f_{1}$ in (17), an RBFNN is introduced into approximate it, then one has

18 $$f_{1} = (w_{1}^{*} )^{T} \psi_{1} (X_{1} ) + \varepsilon_{1} (X_{1} ),\;\left| {\varepsilon_{1} (X_{1} )} \right| \le \Xi_{1}$$

where $\varepsilon_{1} (X_{1} )$ is approximation error, $\Xi_{1} \ge 0$ represents an unknown positive constant. For the ease of future analysis, these variables $X_{i}$, $i = 1, ldots ,n$, will be ignored.

Let $\Theta_{1} = \left[ {\vartheta_{1}^{T} ,(w_{1}^{*} )^{T} ,\dot{y}_{d} } \right]^{T}$ and $\Phi_{1} = \left[ {\phi_{1}^{T} ,\psi_{1}^{T} , - 1} \right]^{T}$, and substituting (18) into (17) gets

19 $$\dot{V}_{11} = z_{1} (x_{2}^{{\rho_{1} }} - \upsilon_{2}^{{\rho_{1} }} ) + z_{1} \upsilon_{2}^{{\rho_{1} }} + z_{1} \Theta_{1}^{T} \Phi_{1} + z_{1} \varepsilon_{1}$$

Applying Lemmas 3, 5 and 6, we have

20 $$\left| {z_{1} (x_{2}^{{\rho_{1} }} - \upsilon_{2}^{{\rho_{1} }} )} \right| \le \rho_{1} \left| {z_{1} } \right|\left| {z_{2} } \right|\left( {x_{2}^{{(\rho_{1} - 1)}} + \upsilon_{2}^{{(\rho_{1} - 1)}} } \right) \le \frac{1}{2}z_{1}^{2} + \frac{1}{2}\rho_{1}^{2} z_{2}^{2} \left( {x_{2}^{{(\rho_{1} - 1)}} + \upsilon_{2}^{{(\rho_{1} - 1)}} } \right)^{2}$$

21 $$\left| {z_{1} \Theta_{1}^{T} \Phi_{1} } \right| \le \frac{1}{{2a_{1}^{2} }}z_{1}^{2} \Upsilon_{1} \Gamma_{1} + \frac{1}{2}a_{1}^{2}$$

22 $$\left| {z_{1} \varepsilon_{1} } \right| \le \Xi_{1} \left| {z_{1} } \right| \le \frac{{\Xi_{1} z_{1}^{2} }}{{\sqrt {z_{1}^{2} + \eta_{1}^{2} } }} + \Xi_{1} \eta_{1}$$

where $\Upsilon_{1} = (\Theta_{1} )^{T} \Theta_{1}$ and $\Gamma_{1} = (\Phi_{1} )^{T} \Phi_{1}$, $a_{1} > 0$ and $\eta_{1} > 0$ represent design parameters.

Substituting (20)–(22) into (19), we have

23 $$\dot{V}_{11} \le z_{1} \upsilon_{2}^{{\rho_{1} }} + \frac{1}{2}z_{1}^{2} + \frac{1}{2}\rho_{1}^{2} z_{2}^{2} \left( {x_{2}^{{(\rho_{1} - 1)}} + \upsilon_{2}^{{(\rho_{1} - 1)}} } \right)^{2} + \frac{1}{{2a_{1}^{2} }}z_{1}^{2} \Upsilon_{1} \Gamma_{1} + \frac{{\Xi_{1} z_{1}^{2} }}{{\sqrt {z_{1}^{2} + \eta_{1}^{2} } }} + \frac{1}{2}a_{1}^{2} + \Xi_{1} \eta_{1}$$

Construct the following Lyapunov function

24 $$V_{1} = V_{11} + \frac{1}{{2\gamma_{1} }}\tilde{\Upsilon }_{1}^{2} + \frac{1}{{2\lambda_{1} }}\tilde{\Xi }_{1}^{2}$$

where $\gamma_{1} > 0$ and $\lambda_{1} > 0$ represent design parameters, $\tilde{\Upsilon }_{1} = \Upsilon_{1} - \hat{\Upsilon }_{1}$ and $\tilde{\Xi }_{1} = \Xi_{1} - \hat{\Xi }_{1}$, $\hat{\Upsilon }_{1}$ and $\hat{\Xi }_{1}$ represent the estimations of $\Upsilon_{1}$ and $\Xi_{1}$, respectively.

Design the adaptive control laws $\hat{\Upsilon }_{1}$ and $\hat{\Xi }_{1}$, and the virtual control law $\upsilon_{2}$ as

25 $$\dot{\hat{\Upsilon }}_{1} = \frac{{\gamma_{1} }}{{2a_{1}^{2} }}z_{1}^{2} \Gamma_{1} - \theta_{11} \hat{\Upsilon }_{1} - \theta_{12} \hat{\Upsilon }_{1}^{(2p - 1)}$$

26 $$\dot{\hat{\Xi }}_{1} = \frac{{\lambda_{1} z_{1}^{2} }}{{\sqrt {z_{1}^{2} + \eta_{1}^{2} } }} - \varphi_{11} \hat{\Xi }_{1} - \varphi_{12} \hat{\Xi }_{1}^{(2p - 1)}$$

27 $$\upsilon_{2} = - \left[ {c_{11} z_{1}^{(2q - 1)} + c_{12} z_{1}^{(2p - 1)} + \frac{1}{2}z_{1} + \frac{1}{{2a_{1}^{2} }}z_{1} \hat{\Upsilon }_{1} \Gamma_{1} + \frac{{\hat{\Xi }_{1} z_{1} }}{{\sqrt {z_{1}^{2} + \eta_{1}^{2} } }}} \right]^{{\frac{1}{{\rho_{1} }}}}$$

where $0 < q < 1$, $p > 1$, $\theta_{11} > 0$, $\theta_{12} > 0$, $\varphi_{11} > 0$, $\varphi_{12} > 0$, $c_{11} > 0$ and $c_{12} > 0$ are design parameters.

Taking the derivative of $V_{1}$ and considering (23) and (25)–(27), we get

28 $$\begin{aligned} & \dot{V}_{1} = \dot{V}_{11} - \frac{1}{{\gamma_{1} }}\tilde{\Upsilon }_{1} \dot{\hat{\Upsilon }}_{1} - \frac{1}{{\lambda_{1} }}\tilde{\Xi }_{1} \dot{\hat{\Xi }}_{1} \\ & \quad \le - c_{11} z_{1}^{2q} - c_{12} z_{1}^{2p} + \frac{{\theta_{11} }}{{\gamma_{1} }}\tilde{\Upsilon }_{1} \hat{\Upsilon }_{1} + \frac{{\varphi_{11} }}{{\lambda_{1} }}\tilde{\Xi }_{1} \hat{\Xi }_{1} + \frac{{\theta_{12} }}{{\gamma_{1} }}\tilde{\Upsilon }_{1} \hat{\Upsilon }_{1}^{(2p - 1)} + \frac{{\varphi_{12} }}{{\lambda_{1} }}\tilde{\Xi }_{1} \hat{\Xi }_{1}^{(2p - 1)} \\ & \quad \quad + \frac{1}{2}\rho_{1}^{2} z_{2}^{2} \left( {x_{2}^{{(\rho_{1} - 1)}} + \upsilon_{2}^{{(\rho_{1} - 1)}} } \right)^{2} + \left( {\frac{1}{2}a_{1}^{2} + \Xi_{1} \eta_{1} } \right) \\ \end{aligned}$$

**Step **$i$($i = 2, \ldots ,n - 1$): Noting the subsystem $\dot{x}_{i} = x_{i + 1}^{{\rho_{i} }} + \vartheta_{i}^{T} \phi_{i} + f_{i}$ and $z_{i} = x_{i} - \upsilon_{i}$, then the derivative of $z_{i}$ as

29 $$\dot{z}_{i} = (x_{i + 1}^{{\rho_{i} }} - \upsilon_{i + 1}^{{\rho_{i} }} ) + \upsilon_{i + 1}^{{\rho_{i} }} + \vartheta_{i}^{T} \phi_{i} + f_{i} - \dot{\upsilon }_{i}$$

Let $V_{i1} = V_{i - 1} {{ + z_{i}^{2} } \mathord{\left/ {\vphantom {{ + z_{i}^{2} } 2}} \right. \kern-0pt} 2}$, the derivative of $V_{i1}$ is

30 $$\dot{V}_{i1} = \dot{V}_{i - 1} + z_{i} (x_{i + 1}^{{\rho_{i} }} - \upsilon_{i + 1}^{{\rho_{i} }} ) + z_{i} \upsilon_{i + 1}^{{\rho_{i} }} + z_{i} \left( {\vartheta_{i}^{T} \phi_{i} + f_{i} - \dot{\upsilon }_{i} } \right)$$

According to the $(i - 1){\text{th}}$ step, it is easy to obtain $\dot{V}_{i - 1}$ as

31 $$\begin{gathered} \dot{V}_{i - 1} \le - \sum\limits_{k = 1}^{i - 1} {c_{k1} z_{k}^{2q} } - \sum\limits_{k = 1}^{i - 1} {c_{k2} z_{k}^{2p} } + \sum\limits_{k = 1}^{i - 1} {\frac{{\theta_{k1} }}{{\gamma_{k} }}\tilde{\Upsilon }_{k} \hat{\Upsilon }_{k} } + \sum\limits_{k = 1}^{i - 1} {\frac{{\varphi_{k1} }}{{\lambda_{k} }}\tilde{\Xi }_{k} \hat{\Xi }_{k} } + \sum\limits_{k = 1}^{i - 1} {\frac{{\theta_{k2} }}{{\gamma_{k} }}\tilde{\Upsilon }_{k} \hat{\Upsilon }_{k}^{(2p - 1)} } \hfill \\ \quad \quad \; + \sum\limits_{k = 1}^{i - 1} {\frac{{\varphi_{k2} }}{{\lambda_{k} }}\tilde{\Xi }_{k} \hat{\Xi }_{k}^{(2p - 1)} } + \frac{1}{2}\rho_{i - 1}^{2} z_{i}^{2} \left( {x_{i}^{{(\rho_{i - 1} - 1)}} + \upsilon_{i}^{{(\rho_{i - 1} - 1)}} } \right)^{2} + \sum\limits_{k = 1}^{i - 1} {\left( {\frac{1}{2}a_{k}^{2} + \Xi_{k} \eta_{k} } \right)} \hfill \\ \end{gathered}$$

Let $F_{i} = f_{i} + \frac{1}{2}\rho_{i - 1}^{2} z_{i} \left( {x_{i}^{{(\rho_{i - 1} - 1)}} + \upsilon_{i}^{{(\rho_{i - 1} - 1)}} } \right)^{2}$, thus, an RBFNN is introduced to approximate $F_{i}$, then one has

32 $$F_{i} = (w_{i}^{*} )^{T} \psi_{i} + \varepsilon_{i} ,\;\left| {\varepsilon_{i} } \right| \le \Xi_{i}$$

where $\varepsilon_{i}$ is approximation error, $\Xi_{i} \ge 0$ represents an unknown positive constant.

Let $\Theta_{i} = \left[ {\vartheta_{i}^{T} ,(w_{i}^{*} )^{T} ,\dot{\upsilon }_{i} } \right]^{T}$ and $\Phi_{i} = \left[ {\phi_{i}^{T} ,\psi_{i}^{T} , - 1} \right]^{T}$, and applying Lemmas 3, 5 and 6, we obtain

33 $$\left| {z_{i} (x_{i + 1}^{{\rho_{i} }} - \upsilon_{i + 1}^{{\rho_{i} }} )} \right| \le \frac{1}{2}z_{i}^{2} + \frac{1}{2}\rho_{i}^{2} z_{i + 1}^{2} \left( {x_{i + 1}^{{(\rho_{i} - 1)}} + \upsilon_{i + 1}^{{(\rho_{i} - 1)}} } \right)^{2}$$

34 $$\left| {z_{i} \Theta_{i}^{T} \Phi_{i} } \right| \le \frac{1}{{2a_{i}^{2} }}z_{i}^{2} \Upsilon_{i} \Gamma_{i} + \frac{1}{2}a_{i}^{2}$$

35 $$\left| {z_{i} \varepsilon_{i} } \right| \le \frac{{\Xi_{i} z_{i}^{2} }}{{\sqrt {z_{i}^{2} + \eta_{i}^{2} } }} + \Xi_{i} \eta_{i}$$

where $\Upsilon_{i} = (\Theta_{i} )^{T} \Theta_{i}$ and $\Gamma_{i} = (\Phi_{i} )^{T} \Phi_{i}$, $a_{i} > 0$ and $\eta_{i} > 0$ represent design parameters.

Substituting (31)–(35) into (30), one has

36 $$\begin{aligned} & \dot{V}_{i1} \le - \sum\limits_{k = 1}^{i - 1} {c_{k1} z_{k}^{2q} } - \sum\limits_{k = 1}^{i - 1} {c_{k2} z_{k}^{2p} } + \sum\limits_{k = 1}^{i - 1} {\frac{{\theta_{k1} }}{{\gamma_{k} }}\tilde{\Upsilon }_{k} \hat{\Upsilon }_{k} } + \sum\limits_{k = 1}^{i - 1} {\frac{{\varphi_{k1} }}{{\lambda_{k} }}\tilde{\Xi }_{k} \hat{\Xi }_{k} } + \sum\limits_{k = 1}^{i - 1} {\frac{{\theta_{k2} }}{{\gamma_{k} }}\tilde{\Upsilon }_{k} \hat{\Upsilon }_{k}^{(2p - 1)} } \\ & \quad + \sum\limits_{k = 1}^{i - 1} {\frac{{\varphi_{k2} }}{{\lambda_{k} }}\tilde{\Xi }_{k} \hat{\Xi }_{k}^{(2p - 1)} } + \frac{1}{2}z_{i}^{2} + \frac{1}{2}\rho_{i}^{2} z_{i + 1}^{2} \left( {x_{i + 1}^{{(\rho_{i} - 1)}} + \upsilon_{i + 1}^{{(\rho_{i} - 1)}} } \right)^{2} + z_{i} \upsilon_{i + 1}^{{\rho_{i} }} \\ & \quad + \frac{1}{{2a_{i}^{2} }}z_{i}^{2} \Upsilon_{i} \Gamma_{i} + \frac{{\Xi_{i} z_{i}^{2} }}{{\sqrt {z_{i}^{2} + \eta_{i}^{2} } }} + \sum\limits_{k = 1}^{i} {\left( {\frac{1}{2}a_{k}^{2} + \Xi_{k} \eta_{k} } \right)} \\ \end{aligned}$$

Construct the following Lyapunov function

37 $$V_{i} = V_{i1} + \frac{1}{{2\gamma_{i} }}\tilde{\Upsilon }_{i}^{2} + \frac{1}{{2\lambda_{i} }}\tilde{\Xi }_{i}^{2}$$

where $\gamma_{i} > 0$ and $\lambda_{i} > 0$ represent design parameters, $\tilde{\Upsilon }_{i} = \Upsilon_{i} - \hat{\Upsilon }_{i}$ and $\tilde{\Xi }_{i} = \Xi_{i} - \hat{\Xi }_{i}$, $\hat{\Upsilon }_{i}$ and $\hat{\Xi }_{i}$ represent the estimations of $\Upsilon_{i}$ and $\Xi_{i}$, respectively.

Design the adaptive control laws $\hat{\Upsilon }_{i}$ and $\hat{\Xi }_{i}$, and the virtual control law $\upsilon_{i + 1}$ as

38 $$\dot{\hat{\Upsilon }}_{i} = \frac{{\gamma_{i} }}{{2a_{i}^{2} }}z_{i}^{2} \Gamma_{i} - \theta_{i1} \hat{\Upsilon }_{i} - \theta_{i2} \hat{\Upsilon }_{i}^{(2p - 1)}$$

39 $$\dot{\hat{\Xi }}_{i} = \frac{{\lambda_{i} z_{i}^{2} }}{{\sqrt {z_{i}^{2} + \eta_{i}^{2} } }} - \varphi_{i1} \hat{\Xi }_{i} - \varphi_{i2} \hat{\Xi }_{i}^{(2p - 1)}$$

40 $$\upsilon_{i + 1} = - \left[ {c_{i1} z_{i}^{(2q - 1)} + c_{i2} z_{i}^{(2p - 1)} + \frac{1}{2}z_{i} + \frac{1}{{2a_{i}^{2} }}z_{i} \hat{\Upsilon }_{i} \Gamma_{i} + \frac{{\hat{\Xi }_{i} z_{i} }}{{\sqrt {z_{i}^{2} + \eta_{i}^{2} } }}} \right]^{{\frac{1}{{\rho_{i} }}}}$$

where $\theta_{i1} > 0$, $\theta_{i2} > 0$, $\varphi_{i1} > 0$, $\varphi_{i2} > 0$, $c_{i1} > 0$ and $c_{i2} > 0$ are design parameters.

Taking the derivative of $V_{i}$ and considering (36) and (38)–(40), we have

41 $$\begin{gathered} \dot{V}_{i} \le - \sum\limits_{k = 1}^{i} {c_{k1} z_{k}^{2q} } - \sum\limits_{k = 1}^{i} {c_{k2} z_{k}^{2p} } + \sum\limits_{k = 1}^{i} {\frac{{\theta_{k1} }}{{\gamma_{k} }}\tilde{\Upsilon }_{k} \hat{\Upsilon }_{k} } + \sum\limits_{k = 1}^{i} {\frac{{\varphi_{k1} }}{{\lambda_{k} }}\tilde{\Xi }_{k} \hat{\Xi }_{k} } + \sum\limits_{k = 1}^{i} {\frac{{\theta_{k2} }}{{\gamma_{k} }}\tilde{\Upsilon }_{k} \hat{\Upsilon }_{k}^{(2p - 1)} } \hfill \\ \quad \;\;\; + \sum\limits_{k = 1}^{i} {\frac{{\varphi_{k2} }}{{\lambda_{k} }}\tilde{\Xi }_{k} \hat{\Xi }_{k}^{(2p - 1)} } + \frac{1}{2}\rho_{i}^{2} z_{i + 1}^{2} \left( {x_{i + 1}^{{(\rho_{i} - 1)}} + \upsilon_{i + 1}^{{(\rho_{i} - 1)}} } \right)^{2} + \sum\limits_{k = 1}^{i} {\left( {\frac{1}{2}a_{k}^{2} + \Xi_{k} \eta_{k} } \right)} \hfill \\ \end{gathered}$$

**Step **$n$: In this step, the adaptive NN-based FTTC law is proposed. Noting the subsystem $\dot{x}_{n} = \left( {{\mathcal{D}}(u)} \right)^{{\rho_{n} }} + \vartheta_{n}^{T} \phi_{n} + f_{n}$, ${\mathcal{D}}(u) = \kappa_{1} (t)u(t) + \kappa_{2} (t)$, $z_{n} = x_{n} - \upsilon_{n}$ and Lemma 4, then the derivative of $z_{n}$ as

42 $$\dot{z}_{n} \le G(t)\left( {u(t)} \right)^{{\rho_{n} }} + \ell \left( {\kappa_{2} (t)} \right)^{{\rho_{n} }} + \vartheta_{n}^{T} \phi_{n} + f_{n} - \dot{\upsilon }_{n}$$

where $G(t) = \hbar \left( {\kappa_{1} (t)} \right)^{{\rho_{n} }}$ are unknown but bounded constant.

Let $V_{n1} = V_{n - 1} {{ + z_{n}^{2} } \mathord{\left/ {\vphantom {{ + z_{n}^{2} } 2}} \right. \kern-0pt} 2}$, the derivative of $V_{n1}$ is

43 $$\dot{V}_{n1} \le \dot{V}_{n - 1} + G(t)z_{n} \left( {u(t)} \right)^{{\rho_{n} }} + z_{n} \left( {\vartheta_{n}^{T} \phi_{n} + f_{n} + \ell \left( {\kappa_{2} (t)} \right)^{{\rho_{n} }} - \dot{\upsilon }_{n} } \right)$$

Similarly, according to the $(n - 1){\text{th}}$ step, it is easy to obtain $\dot{V}_{n - 1}$ as

44 $$\begin{aligned} & \dot{V}_{i - 1} \le - \sum\limits_{k = 1}^{n - 1} {c_{k1} z_{k}^{2q} } - \sum\limits_{k = 1}^{n - 1} {c_{k2} z_{k}^{2p} } + \sum\limits_{k = 1}^{n - 1} {\frac{{\theta_{k1} }}{{\gamma_{k} }}\tilde{\Upsilon }_{k} \hat{\Upsilon }_{k} } + \sum\limits_{k = 1}^{n - 1} {\frac{{\varphi_{k1} }}{{\lambda_{k} }}\tilde{\Xi }_{k} \hat{\Xi }_{k} } + \sum\limits_{k = 1}^{n - 1} {\frac{{\theta_{k2} }}{{\gamma_{k} }}\tilde{\Upsilon }_{k} \hat{\Upsilon }_{k}^{(2p - 1)} } \\ & \quad + \sum\limits_{k = 1}^{n - 1} {\frac{{\varphi_{k2} }}{{\lambda_{k} }}\tilde{\Xi }_{k} \hat{\Xi }_{k}^{(2p - 1)} } + \frac{1}{2}\rho_{n - 1}^{2} z_{n}^{2} \left( {x_{n}^{{(\rho_{n - 1} - 1)}} + \upsilon_{n}^{{(\rho_{n - 1} - 1)}} } \right)^{2} + \sum\limits_{k = 1}^{n - 1} {\left( {\frac{1}{2}a_{k}^{2} + \Xi_{k} \eta_{k} } \right)} \\ \end{aligned}$$

Let $F_{n} = f_{n} + \ell \left( {\kappa_{2} (t)} \right)^{{\rho_{n} }} + \frac{1}{2}\rho_{n - 1}^{2} z_{n} \left( {x_{n}^{{(\rho_{n - 1} - 1)}} + \upsilon_{n}^{{(\rho_{n - 1} - 1)}} } \right)^{2}$, similarly, an RBFNN is introduced to approximate $F_{n}$, then one gets

45 $$F_{n} = (w_{n}^{*} )^{T} \psi_{n} + \varepsilon_{n} ,\;\left| {\varepsilon_{n} } \right| \le \Xi_{n}$$

where $\varepsilon_{n}$ is approximation error, $\Xi_{n} \ge 0$ represents an unknown positive constant.

Let $\Theta_{n} = \left[ {\vartheta_{n}^{T} ,(w_{n}^{*} )^{T} ,\dot{\upsilon }_{n} } \right]^{T}$ and $\Phi_{n} = \left[ {\phi_{n}^{T} ,\psi_{n}^{T} , - 1} \right]^{T}$, and applying Lemmas 5 and 6, we have

46 $$\left| {z_{n} \Theta_{n}^{T} \Phi_{n} } \right| \le \frac{1}{{2a_{n}^{2} }}z_{n}^{2} \Upsilon_{n} \Gamma_{n} + \frac{1}{2}a_{n}^{2}$$

47 $$\left| {z_{n} \varepsilon_{n} } \right| \le \frac{{\Xi_{n} z_{n}^{2} }}{{\sqrt {z_{n}^{2} + \eta_{n}^{2} } }} + \Xi_{n} \eta_{n}$$

where $\Upsilon_{n} = (\Theta_{n} )^{T} \Theta_{n}$ and $\Gamma_{n} = (\Phi_{n} )^{T} \Phi_{n}$, $a_{n} > 0$ and $\eta_{n} > 0$ represent design parameters.

Substituting (44)–(47) into (43) yields

48 $$\begin{aligned} & \dot{V}_{n1} \le - \sum\limits_{k = 1}^{n - 1} {c_{k1} z_{k}^{2q} } - \sum\limits_{k = 1}^{n - 1} {c_{k2} z_{k}^{2p} } + \sum\limits_{k = 1}^{n - 1} {\frac{{\theta_{k1} }}{{\gamma_{k} }}\tilde{\Upsilon }_{k} \hat{\Upsilon }_{k} } + \sum\limits_{k = 1}^{n - 1} {\frac{{\varphi_{k1} }}{{\lambda_{k} }}\tilde{\Xi }_{k} \hat{\Xi }_{k} } + \sum\limits_{k = 1}^{n - 1} {\frac{{\theta_{k2} }}{{\gamma_{k} }}\tilde{\Upsilon }_{k} \hat{\Upsilon }_{k}^{(2p - 1)} } \\ & \quad + \sum\limits_{k = 1}^{n - 1} {\frac{{\varphi_{k2} }}{{\lambda_{k} }}\tilde{\Xi }_{k} \hat{\Xi }_{k}^{(2p - 1)} } + \frac{1}{{2a_{n}^{2} }}z_{n}^{2} \Upsilon_{n} \Gamma_{n} + \frac{{\Xi_{n} z_{n}^{2} }}{{\sqrt {z_{n}^{2} + \eta_{n}^{2} } }} + G(t)z_{n} \left( {u(t)} \right)^{{\rho_{n} }} \\ & \quad + \sum\limits_{k = 1}^{n} {\left( {\frac{1}{2}a_{k}^{2} + \Xi_{k} \eta_{k} } \right)} \\ \end{aligned}$$

Construct the following Lyapunov function

49 $$V_{n} = V_{n1} + \frac{1}{{2\gamma_{n} }}\tilde{\Upsilon }_{n}^{2} + \frac{1}{{2\lambda_{n} }}\tilde{\Xi }_{n}^{2}$$

where $\gamma_{n} > 0$ and $\lambda_{n} > 0$ are design parameters, $\tilde{\Upsilon }_{n} = \Upsilon_{n} - \hat{\Upsilon }_{n}$ and $\tilde{\Xi }_{n} = \Xi_{n} - \hat{\Xi }_{n}$, $\hat{\Upsilon }_{n}$ and $\hat{\Xi }_{n}$ are the estimations of $\Upsilon_{n}$ and $\Xi_{n}$, respectively.

Since $G(t)$ is an unknown but bounded parameter, the Nussbaum-type function ${\mathcal{N}}(\chi_{n} ) = \exp (\chi_{n}^{2} )\cos (\pi \chi_{n} /2)$ is introduced in the actual control law design. Thus, the adaptive control laws $\hat{\Upsilon }_{n}$, $\hat{\Xi }_{n}$ and $\chi_{n}$, and the actual adaptive NN-based FTTC law $u(t)$ are designed as

50 $$\dot{\hat{\Upsilon }}_{n} = \frac{{\gamma_{n} }}{{2a_{n}^{2} }}z_{n}^{2} \Gamma_{n} - \theta_{n1} \hat{\Upsilon }_{n} - \theta_{n2} \hat{\Upsilon }_{n}^{(2p - 1)}$$

51 $$\dot{\hat{\Xi }}_{n} = \frac{{\lambda_{n} z_{n}^{2} }}{{\sqrt {z_{n}^{2} + \eta_{n}^{2} } }} - \varphi_{n1} \hat{\Xi }_{n} - \varphi_{n2} \hat{\Xi }_{n}^{(2p - 1)}$$

52 $$\dot{\chi }_{n} = z_{n} \beta_{n} (t)$$

53 $$u(t) = \left( {{\mathcal{N}}(\chi_{n} )\beta_{n} (t)} \right)^{{\frac{1}{{\rho_{n} }}}}$$

54 $$\beta_{n} (t) = c_{n1} z_{n}^{(2q - 1)} + c_{n2} z_{n}^{(2p - 1)} + \frac{1}{{2a_{n}^{2} }}z_{n} \hat{\Upsilon }_{n} \Gamma_{n} + \frac{{\hat{\Xi }_{n} z_{n} }}{{\sqrt {z_{n}^{2} + \eta_{n}^{2} } }}$$

where $\theta_{n1} > 0$, $\theta_{n2} > 0$, $\varphi_{n1} > 0$, $\varphi_{n2} > 0$, $c_{n1} > 0$ and $c_{n2} > 0$ are design parameters.

Taking the derivative of $V_{n}$ and considering (49) and (50)–(54), we have

55 $$\begin{aligned} & \dot{V}_{n} \le - \sum\limits_{k = 1}^{n} {c_{k1} z_{k}^{2q} } - \sum\limits_{k = 1}^{n} {c_{k2} z_{k}^{2p} } + \sum\limits_{k = 1}^{n} {\frac{{\theta_{k1} }}{{\gamma_{k} }}\tilde{\Upsilon }_{k} \hat{\Upsilon }_{k} } + \sum\limits_{k = 1}^{n} {\frac{{\varphi_{k1} }}{{\lambda_{k} }}\tilde{\Xi }_{k} \hat{\Xi }_{k} } + \sum\limits_{k = 1}^{n} {\frac{{\theta_{k2} }}{{\gamma_{k} }}\tilde{\Upsilon }_{k} \hat{\Upsilon }_{k}^{(2p - 1)} } \\ & \quad + \sum\limits_{k = 1}^{n} {\frac{{\varphi_{k2} }}{{\lambda_{k} }}\tilde{\Xi }_{k} \hat{\Xi }_{k}^{(2p - 1)} } + \left( {G(t){\mathcal{N}}(\chi_{n} ) + 1} \right)\dot{\chi }_{n} + \sum\limits_{k = 1}^{n} {\left( {\frac{1}{2}a_{k}^{2} + \Xi_{k} \eta_{k} } \right)} \\ \end{aligned}$$

### Stability analysis

Based on the preceding discussion, the primary results can be encapsulated in the following Theorem 1.

#### Theorem 1

Consider the uncertain HONSs (1) with the desired trajectory $y_{d}$ under Assumptions 1 and 2. By designing adaptive control laws (25), (26), (38), (39), (50), (51) and (52), virtual control laws (27) and (40), and the adaptive NN-based FTTC law (53), it can be ensured that.

i. All the signals of the closed-loop system remain bounded.
ii. The tracking error $z_{1}$ satisfies that $\left| {z_{1} } \right| \le \Delta_{0}$ within the fixed time $T_{s}$, where $\Delta_{0}$ and $T_{s}$ are given as
  56 $$\Delta_{0} = \min \left\{ {\left( {\frac{{2^{q} A_{0} }}{\alpha (1 - \varsigma )}} \right)^{{{1 \mathord{\left/ {\vphantom {1 {2q}}} \right. \kern-0pt} {2q}}}} ,\left( {\frac{{2^{p} A_{0} }}{\beta (1 - \varsigma )}} \right)^{{{1 \mathord{\left/ {\vphantom {1 {2p}}} \right. \kern-0pt} {2p}}}} } \right\}$$
  57 $$T_{s} \le T_{\max } : = \frac{1}{\alpha \varsigma (1 - q)} + \frac{1}{\beta \varsigma (p - 1)}$$

where $\alpha$, $\beta$, $\varsigma$ and $A_{0}$ are positive design parameters.

#### Proof

Noting (55) and using Lemma 5, we get

58 $$\frac{{\theta_{k1} }}{{\gamma_{k} }}\tilde{\Upsilon }_{k} \hat{\Upsilon }_{k} \le - \frac{{\theta_{k1} }}{{\gamma_{k} }}\tilde{\Upsilon }_{k}^{2} + \frac{{\theta_{k1} }}{{\gamma_{k} }}\Upsilon_{k}^{2}$$

59 $$\frac{{\varphi_{k1} }}{{\lambda_{k} }}\tilde{\Xi }_{k} \hat{\Xi }_{k} \le - \frac{{\varphi_{k1} }}{{\lambda_{k} }}\tilde{\Xi }_{k}^{2} + \frac{{\varphi_{k1} }}{{\lambda_{k} }}\Xi_{k}^{2}$$

In view of the result of Sun et al.^39^, if the initial states satisfy that $\hat{\Upsilon }_{k} (0) \ge 0$ and $\hat{\Xi }_{k} (0) \ge 0$, then $\hat{\Upsilon }_{k} (t) \ge 0$ and $\hat{\Xi }_{k} (t) \ge 0$ for $\forall t \ge 0$, $k = 1, \ldots ,n$. According to $\hat{\Upsilon }_{k} = \Upsilon_{k} - \tilde{\Upsilon }_{k}$ and $\hat{\Xi }_{k} = \Xi_{k} - \tilde{\Xi }_{k}$, then it is further obtained that $\Upsilon_{k} \ge \tilde{\Upsilon }_{k}$ and $\Xi_{k} \ge \tilde{\Xi }_{k}$. Thereby, we have

60 $$\frac{{\theta_{k2} }}{{\gamma_{k} }}\tilde{\Upsilon }_{k} \hat{\Upsilon }_{k}^{(2p - 1)} \le \frac{2p - 1}{p}\frac{{\theta_{k2} }}{{2\gamma_{k} }}\left( {\Upsilon_{k}^{2p} - \tilde{\Upsilon }_{k}^{2p} } \right)$$

61 $$\frac{{\varphi_{k2} }}{{\lambda_{k} }}\tilde{\Xi }_{k} \hat{\Xi }_{k}^{(2p - 1)} \le \frac{2p - 1}{p}\frac{{\varphi_{k2} }}{{2\lambda_{k} }}\left( {\Xi_{k}^{2p} - \tilde{\Xi }_{k}^{2p} } \right)$$

Substituting (58)–(61) into (55) yields

62 $$\begin{aligned} & \dot{V}_{n} \le - \sum\limits_{k = 1}^{n} {c_{k1} z_{k}^{2q} } - \sum\limits_{k = 1}^{n} {c_{k2} z_{k}^{2p} } - \sum\limits_{k = 1}^{n} {\frac{{\theta_{k1} }}{{2\gamma_{k} }}\tilde{\Upsilon }_{k}^{2} } - \sum\limits_{k = 1}^{n} {\frac{{\varphi_{k1} }}{{2\lambda_{k} }}\tilde{\Xi }_{k}^{2} } - \frac{2p - 1}{p}\sum\limits_{k = 1}^{n} {\frac{{\theta_{k2} }}{{2\gamma_{k} }}\tilde{\Upsilon }_{k}^{2p} } - \frac{2p - 1}{p}\sum\limits_{k = 1}^{n} {\frac{{\varphi_{k2} }}{{2\lambda_{k} }}\tilde{\Xi }_{k}^{2p} } \\ & \quad + \left( {G(t){\mathcal{N}}(\chi_{n} ) + 1} \right)\dot{\chi }_{n} + \sum\limits_{k = 1}^{n} {\left( {\frac{1}{2}a_{k}^{2} + \Xi_{k} \eta_{k} } \right)} + \sum\limits_{k = 1}^{n} {\frac{{\theta_{k1} }}{{2\gamma_{k} }}\Upsilon_{k}^{2} } + \sum\limits_{k = 1}^{n} {\frac{{\varphi_{k1} }}{{2\lambda_{k} }}\Xi_{k}^{2} } \\ & \quad + \frac{2p - 1}{p}\sum\limits_{k = 1}^{n} {\frac{{\theta_{k2} }}{{2\gamma_{k} }}\Upsilon_{k}^{2p} } + \frac{2p - 1}{p}\sum\limits_{k = 1}^{n} {\frac{{\varphi_{k2} }}{{2\lambda_{k} }}\Xi_{k}^{2p} } \\ \end{aligned}$$

Considering Lemma 7, we have

63 $$\begin{aligned} & \dot{V}_{n} \le - \alpha \left[ {\left( {\sum\limits_{k = 1}^{n} {\frac{{z_{k}^{2} }}{2}} } \right)^{q} + \left( {\sum\limits_{k = 1}^{n} {\frac{1}{{2\gamma_{k} }}\tilde{\Upsilon }_{k}^{2} } } \right)^{q} + \left( {\sum\limits_{k = 1}^{n} {\frac{1}{{2\lambda_{k} }}\tilde{\Xi }_{k}^{2} } } \right)^{q} } \right] + \alpha \left[ {\left( {\sum\limits_{k = 1}^{n} {\frac{1}{{2\gamma_{k} }}\tilde{\Upsilon }_{k}^{2} } } \right)^{q} + \left( {\sum\limits_{k = 1}^{n} {\frac{1}{{2\lambda_{k} }}\tilde{\Xi }_{k}^{2} } } \right)^{q} } \right] \\ & \quad - \nu \left[ {\left( {\sum\limits_{k = 1}^{n} {\frac{{z_{k}^{2} }}{2}} } \right)^{p} + \left( {\sum\limits_{k = 1}^{n} {\frac{1}{{2\gamma_{k} }}\tilde{\Upsilon }_{k}^{2} } } \right)^{p} + \left( {\sum\limits_{k = 1}^{n} {\frac{1}{{2\lambda_{k} }}\tilde{\Xi }_{k}^{2} } } \right)^{p} } \right] - \alpha \left( {\sum\limits_{k = 1}^{n} {\frac{1}{{2\gamma_{k} }}\tilde{\Upsilon }_{k}^{2} } + \sum\limits_{k = 1}^{n} {\frac{1}{{2\lambda_{k} }}\tilde{\Xi }_{k}^{2} } } \right) \\ & \quad + \left( {G(t){\mathcal{N}}(\chi_{n} ) + 1} \right)\dot{\chi }_{n} + \sum\limits_{k = 1}^{n} {\left( {\frac{1}{2}a_{k}^{2} + \Xi_{k} \eta_{k} } \right)} + \sum\limits_{k = 1}^{n} {\frac{{\theta_{k1} }}{{2\gamma_{k} }}\Upsilon_{k}^{2} } + \sum\limits_{k = 1}^{n} {\frac{{\varphi_{k1} }}{{2\lambda_{k} }}\Xi_{k}^{2} } \\ & \quad + \frac{2p - 1}{p}\sum\limits_{k = 1}^{n} {\frac{{\theta_{k2} }}{{2\gamma_{k} }}\Upsilon_{k}^{2p} } + \frac{2p - 1}{p}\sum\limits_{k = 1}^{n} {\frac{{\varphi_{k2} }}{{2\lambda_{k} }}\Xi_{k}^{2p} } \\ \end{aligned}$$

where

$$\begin{aligned} \alpha & = \min \left\{ {2^{q} c_{i1} ,\theta_{i1} ,\varphi_{i1} ,i = 1, \ldots ,n} \right\} \\ \nu & = \min \left\{ {2^{p} (n^{(1 - q)} c_{j2} ),p^{ - 1} (2p - 1)(n^{(1 - q)} \theta_{j2} ),p^{ - 1} (2p - 1)(n^{(1 - q)} \varphi_{j2} ),j = 1, \ldots ,n} \right\} \\ \end{aligned}$$

Further, applying Lemma 5, we have

64 $$\left( {\sum\limits_{k = 1}^{n} {\frac{1}{{2\gamma_{k} }}\tilde{\Upsilon }_{k}^{2} } } \right)^{q} \le \sum\limits_{k = 1}^{n} {\frac{1}{{2\gamma_{k} }}\tilde{\Upsilon }_{k}^{2} } + (1 - q)q^{{\frac{q}{1 - q}}}$$

65 $$\left( {\sum\limits_{k = 1}^{n} {\frac{1}{{2\lambda_{k} }}\tilde{\Xi }_{k}^{2} } } \right)^{q} \le \frac{1}{{2\lambda_{k} }}\tilde{\Xi }_{k}^{2} + (1 - q)q^{{\frac{q}{1 - q}}}$$

Substituting (64) and (65) into (63), and considering Lemma 7, one has

66 $$\begin{aligned} & \dot{V}_{n} \le - \alpha \left[ {\left( {\sum\limits_{k = 1}^{n} {\frac{{z_{k}^{2} }}{2}} } \right)^{q} + \left( {\sum\limits_{k = 1}^{n} {\frac{1}{{2\gamma_{k} }}\tilde{\Upsilon }_{k}^{2} } } \right)^{q} + \left( {\sum\limits_{k = 1}^{n} {\frac{1}{{2\lambda_{k} }}\tilde{\Xi }_{k}^{2} } } \right)^{q} } \right] + \left( {G(t){\mathcal{N}}(\chi_{n} ) + 1} \right)\dot{\chi }_{n} + C_{0} \\ & \quad - \nu \left[ {\left( {\sum\limits_{k = 1}^{n} {\frac{{z_{k}^{2} }}{2}} } \right)^{p} + \left( {\sum\limits_{k = 1}^{n} {\frac{1}{{2\gamma_{k} }}\tilde{\Upsilon }_{k}^{2} } } \right)^{p} + \left( {\sum\limits_{k = 1}^{n} {\frac{1}{{2\lambda_{k} }}\tilde{\Xi }_{k}^{2} } } \right)^{p} } \right] \\ & \quad \le - \alpha V_{n}^{q} - \beta V_{n}^{p} + \left( {G(t){\mathcal{N}}(\chi_{n} ) + 1} \right)\dot{\chi }_{n} + C_{0} \\ \end{aligned}$$

where

$$\begin{aligned} \beta & = (2^{1 - p} )\nu \\ C_{0} & = \sum\limits_{k = 1}^{n} {\left( {\frac{1}{2}a_{k}^{2} + \Xi_{k} \eta_{k} } \right)} + \sum\limits_{k = 1}^{n} {\frac{{\theta_{k1} }}{{2\gamma_{k} }}\Upsilon_{k}^{2} } + \sum\limits_{k = 1}^{n} {\frac{{\varphi_{k1} }}{{2\lambda_{k} }}\Xi_{k}^{2} } + \frac{2p - 1}{p}\sum\limits_{k = 1}^{n} {\frac{{\theta_{k2} }}{{2\gamma_{k} }}\Upsilon_{k}^{2p} } + \frac{2p - 1}{p}\sum\limits_{k = 1}^{n} {\frac{{\varphi_{k2} }}{{2\lambda_{k} }}\Xi_{k}^{2p} } \\ & \quad + 2\alpha (1 - q)q^{{\frac{q}{1 - q}}} \\ \end{aligned}$$

According to the definition of $V_{n}$, we have $V_{n} \ge 0$, then it can be got that $V_{n} \le V_{n}^{q} + V_{n}^{p}$ for $q \in (0,1)$ and $p \in (1, + \infty )$. Therefore, the inequality (66) can be rewritten as

67 $$\dot{V}_{n} \le - C_{1} V_{n} + \left( {G(t){\mathcal{N}}(\chi_{n} ) + 1} \right)\dot{\chi }_{n} + C_{0}$$

where $$C_{1} = \min \left\{ {\alpha ,\beta } \right\}$$.

Multiplying $$e^{{C_{1} t}}$$ on both sides of (67), and integrating $$[0,t)$$, we get

68 $$V_{n} (t) \le e^{{( - C_{1} t)}} \int_{0}^{t} {e^{{(C_{1} \tau )}} \left( {G(\tau ){\mathcal{N}}(\chi_{n} ) + 1} \right)\dot{\chi }_{n} d\tau } + \frac{{C_{0} }}{{C_{1} }} + V_{n} (0)$$

Considering Lemma 1, it can be obtained that $$V_{n} (t)$$, $$\chi_{n} (t)$$ and $$\int_{0}^{t} {e^{{(C_{1} \tau )}} \left( {G(\tau ){\mathcal{N}}(\chi_{n} ) + 1} \right)\dot{\chi }_{n} d\tau }$$ are bounded in $$[0,t)$$. Recalling the definition of $$V_{n}$$, then the boundedness of $$z_{i}$$, $$\tilde{\Upsilon }_{i}$$ and $$\tilde{\Xi }_{i}$$, $$i = 1, \ldots ,n$$, can be guaranteed. Since $$z_{i}$$ and $$\chi_{n}$$ are bounded, it can be guaranteed that $$\hat{\Upsilon }_{i}$$, $$\hat{\Xi }_{i}$$ and $$u(t)$$ are also bounded. Thus, all signals in the closed-loop systems are bounded.

In view of the boundedness of $\int_{0}^{t} {e^{{(C_{1} \tau )}} \left( {G(\tau ){\mathcal{N}}(\chi_{n} ) + 1} \right)\dot{\chi }_{n} d\tau }$, it is further obtained that $\left( {G(t){\mathcal{N}}(\chi_{n} ) + 1} \right)\dot{\chi }_{n}$ is bounded. Without losing generality, let $\left| {\left( {G(t){\mathcal{N}}(\chi_{n} ) + 1} \right)\dot{\chi }_{n} } \right| \le D_{0}$ with $D_{0}$ being a positive constant. Hence, the inequality (66) can be further described as

69 $$\dot{V}_{n} \le - \alpha V_{n}^{q} - \beta V_{n}^{p} + A_{0}$$

where $$A_{0} = C_{0} + D_{0}$$.

Further, considering Lemma 2, it can be easily observed that the system (1) is practically fixed-time stable under the designed control law, and the fixed time is

70 $$T_{s} \le T_{\max } : = \frac{1}{\alpha \varsigma (1 - q)} + \frac{1}{\beta \varsigma (p - 1)}$$

and for $t \ge T_{s}$, the tracking error $z_{1}$ satisfies that

71 $$\left| {z_{1} } \right| \le \min \left\{ {\left( {\frac{{2^{q} A_{0} }}{\alpha (1 - \varsigma )}} \right)^{{{1 \mathord{\left/ {\vphantom {1 {2q}}} \right. \kern-0pt} {2q}}}} ,\left( {\frac{{2^{p} A_{0} }}{\beta (1 - \varsigma )}} \right)^{{{1 \mathord{\left/ {\vphantom {1 {2p}}} \right. \kern-0pt} {2p}}}} } \right\}$$

where $$0 < \varsigma < 1$$.

Observing (71), it can be concluded that by selecting suitable design parameters, the tracking error can converge to a small neighborhood of zero. The proof is completed.□

#### Remark 2

Noting (71), as the values of $\alpha$ and $\beta$ increase or the value of $A_{0}$ decreases, the tracking error $z_{1}$ will gradually decrease. Meanwhile, it can also be seen that $\alpha$, $\beta$ and $A_{0}$ can be changed by adjusting the values of $c_{i1}$, $c_{i2}$, $\theta_{i1}$, $\theta_{i2}$, $\varphi_{i1}$, $\varphi_{i2}$, $\gamma_{i}$, $\lambda_{i}$ and $a_{i}$, $i = 1, \ldots ,n$. Based on (50)–(54), it is evident that the modification of the aforementioned parameters influences the control signal. Consequently, when determining the parameters, it is essential to make appropriate trade-off between the tracking performance and the control signal.

### Simulation analysis

To demonstrate the efficacy of the proposed control law, a type of uncertain HONSs with time-varying parameters is presented as

72 $$\begin{aligned} \dot{x}_{1} & = x_{2}^{5} + \vartheta_{1}^{T} (t)\phi_{1} (\overline{x}_{1} ) + f_{1} (\overline{x}_{1} ) \\ \dot{x}_{2} & = x_{3}^{7} + \vartheta_{2}^{T} (t)\phi_{2} (\overline{x}_{2} ) + f_{2} (\overline{x}_{2} ) \\ \dot{x}_{3} & = \left( {{\mathcal{D}}(u)} \right)^{5} + \vartheta_{3}^{T} (t)\phi_{3} (\overline{x}_{3} ) + f_{3} (\overline{x}_{3} ) \\ \end{aligned}$$

where $$\vartheta_{1} (t) = 2\cos (0.5t)$$, $$\vartheta_{2} (t) = \left[ {\sin (0.5t),1.2} \right]^{T}$$, $$\vartheta_{3} (t) = \left[ {1.5,\sin (t),0.5} \right]^{T}$$, $$\phi_{1} (\overline{x}_{1} ) = \cos (x_{1} )$$, $$\phi_{2} (\overline{x}_{2} ) = \left[ {x_{1} ,\sin (x_{2} )} \right]^{T}$$, $$\phi_{3} (\overline{x}_{3} ) = \left[ {x_{1} x_{2} ,x_{3} ,\sin (x_{2} x_{3} )} \right]^{T}$$, $$f_{1} (\overline{x}_{1} ) = 2.5e^{{( - 0.2x_{1} )}}$$, $$f_{2} (\overline{x}_{2} ) = 3x_{1} x_{2}^{2}$$ and $$f_{3} (\overline{x}_{3} ) = 0.5e^{{ - 2.5x_{2} }} \cos (x_{1} x_{3} )$$.

During the simulation process, the initial conditions of $x_{i} (0)$, $\hat{\Upsilon }_{i} (0)$, $\hat{\Xi }_{i} (0)$, $i = 1,2,3$, and $\chi_{3} (0)$ are given as $x_{1} (0) = 0.2$, $x_{2} (0) = 0.5$, $x_{3} (0) = 0.3$, $\hat{\Upsilon }_{1} (0) = \hat{\Upsilon }_{2} (0) = \hat{\Upsilon }_{3} (0) = 0.01$, $\hat{\Xi }_{1} (0) = \hat{\Xi }_{2} (0) = \hat{\Xi }_{3} (0) = 0.01$ and $\chi_{3} (0) = 0.0$. The desired trajectory is selected as $y_{d} = 1.5\sin (1.5t) + 1.5\sin (2.5t)$, and the simulation is set as $t = 20({\text{s}})$.

The RBFNN for $f_{1}$ contains 9 nodes with the center $\text{B}_{1}$ evenly spaced in $[ - 8,8]$ and the width a_1_=2.0, the RBFNN for $F_{2}$ contains 9 nodes with the center $\text{B}_{2}$ evenly spaced in $[ - 8,8] \times [ - 8,8] \times [ - 8,8]$ and the width a_2_=2.0, and the RBFNN for $F_{3}$ contains 9 nodes with the center $\text{B}_{3}$ evenly spaced in $[ - 8,8] \times [ - 8,8] \times [ - 8,8] \times [ - 8,8]$ and the width a_3_=2.0. Other design parameters are given as $\gamma_{1} = 2.0$, $\gamma_{2} = 1.5$, $\gamma_{3} = 0.25$, $\lambda_{1} = 2.5$, $\lambda_{2} = 0.5$, $\lambda_{3} = 1.5$, $\theta_{11} = 5.5$, $\theta_{21} = 2.5$, $\theta_{31} = 0.05$, $\theta_{12} = 7.5$, $\theta_{22} = 6.5$, $\theta_{32} = 0.15$, $\varphi_{11} = 2.0$, $\varphi_{21} = 5.0$, $\varphi_{31} = 0.05$, $\varphi_{12} = 1.0$, $\varphi_{22} = 4.5$, $\varphi_{32} = 0.15$, $c_{11} = c_{12} = 130$, $c_{21} = c_{22} = 85$, $c_{31} = c_{32} = 3.5$, $q = 0.9$, $p = 1.8$, $a_{1} = a_{2} = a_{3} = 1.0$, and $\eta_{1} = \eta_{2} = \eta_{3} = 1.0$.

The unknown input nonlinearity ${\mathcal{D}}(u)$ is selected for the following three cases.

Case 1

${\mathcal{D}}(u)$ is selected as a type of unknown actuator fault^3^, which is described as

73 $${\mathcal{D}}(u) = \zeta u(t) + b_{0} (t)$$

where $\zeta \in (0,1)$, $b_{0} (t)$ is a bounded time-varying bias signal that occurs after $t_{0}$, that is, there is $\left| {b_{0} (t)} \right| \le b_{0}^{*}$ for $\forall t \ge t_{0}$ with $b_{0}^{*}$ being a positive constant.

**Fig. 1:**
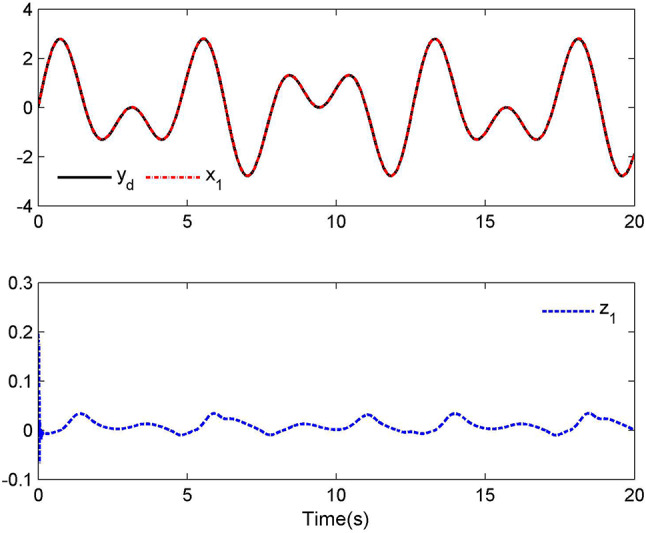
Tracking performance for Case 1.

**Fig. 2:**
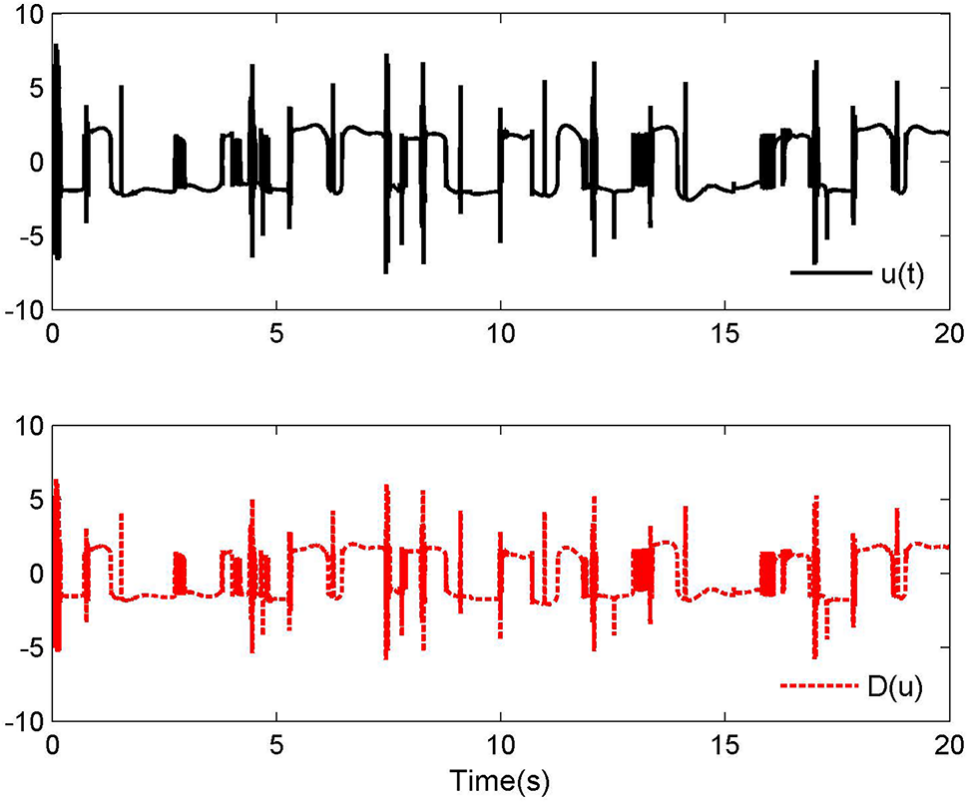
Control laws $u(t)$ and ${\mathcal{D}}(u)$ for Case 1.

**Fig. 3:**
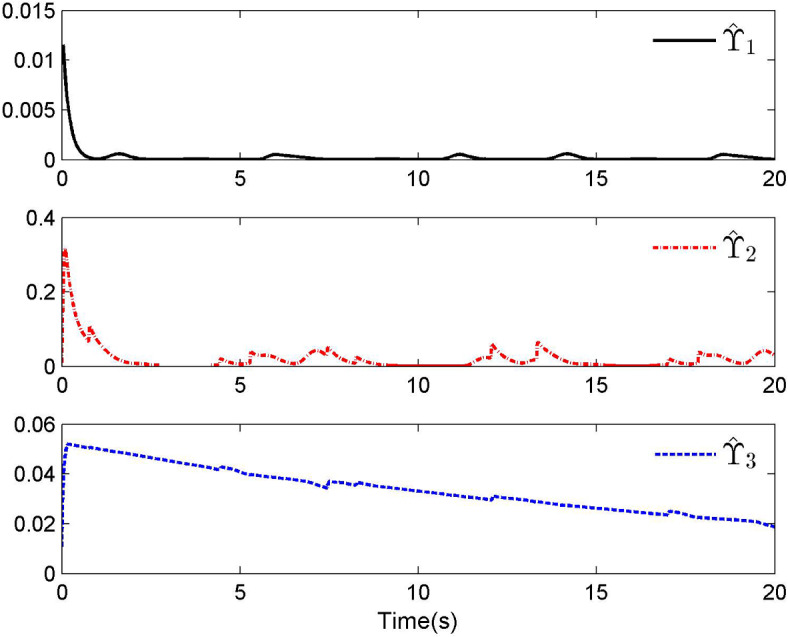
Adaptive control laws $\hat{\Upsilon }_{1}$, $\hat{\Upsilon }_{2}$ and $\hat{\Upsilon }_{3}$ for Case 1.

**Fig. 4:**
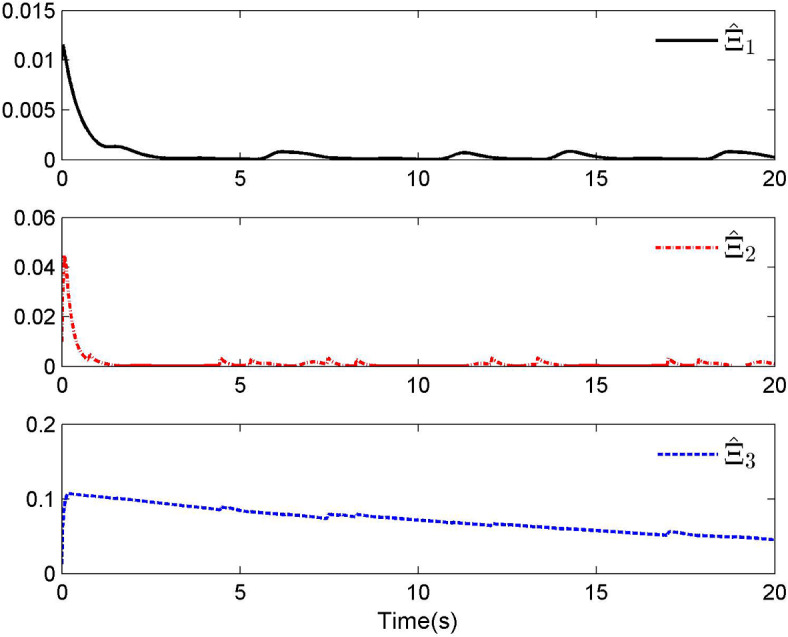
Adaptive control laws $\hat{\Xi }_{1}$, $\hat{\Xi }_{2}$ and $\hat{\Xi }_{3}$ for Case 1.

Compared with (2), we have $\kappa_{1} (t) = \xi$ and $\kappa_{2} (t) = b_{0} (t)$. In this case, let $\kappa_{1} (t) = 0.8$, $\kappa_{2} (t) = 0.25\sin (t)$ and $t_{0} = 4({\text{s)}}$. The simulation results are provided in Figs. 1, 2, 3 and 4.

Case 2

${\mathcal{D}}(u)$ is selected as a type of unknown dead-zone input^12^, which is described as74where $\delta_{l}$ and $\delta_{r}$ are the left and right slope of dead-zone input, $\text{Q}_{l}$ and $\text{Q}_{l}$ are the left and right breakpoints, $\delta_{l} > 0$, $\delta_{r} > 0$, $\text{Q}_{l} > 0$ and $\text{Q}_{r} > 0$ are design parameters.

Compared with (2), we have $\kappa_{1} (t) = \delta$ and $\kappa_{2} (t) = d_{0} (u)$, where $\delta$ and $d_{0} (t)$ satisfy that

75 $$\delta = \left\{ {\begin{array}{*{20}c} {\delta_{l} } & {u(t) \le 0} \\ {\delta_{r} } & {u(t) > 0} \\ \end{array} } \right.$$

**Fig. 5:**
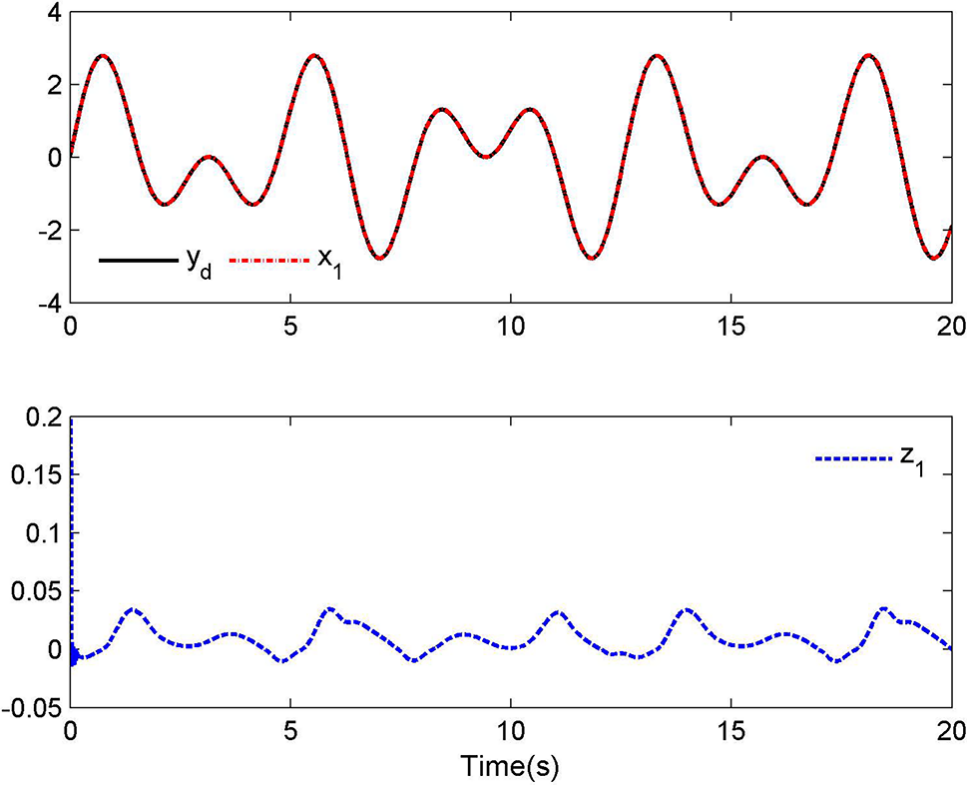
Tracking performance for Case 2.

**Fig. 6:**
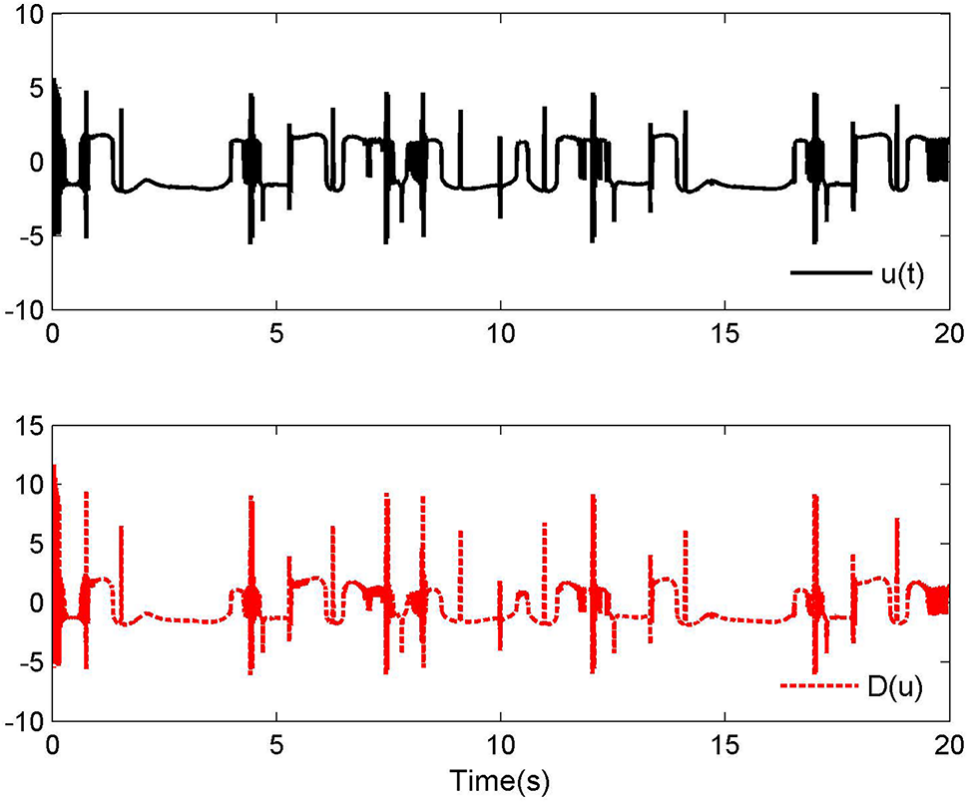
Control laws $u(t)$ and ${\mathcal{D}}(u)$ for Case 2.

**Fig. 7:**
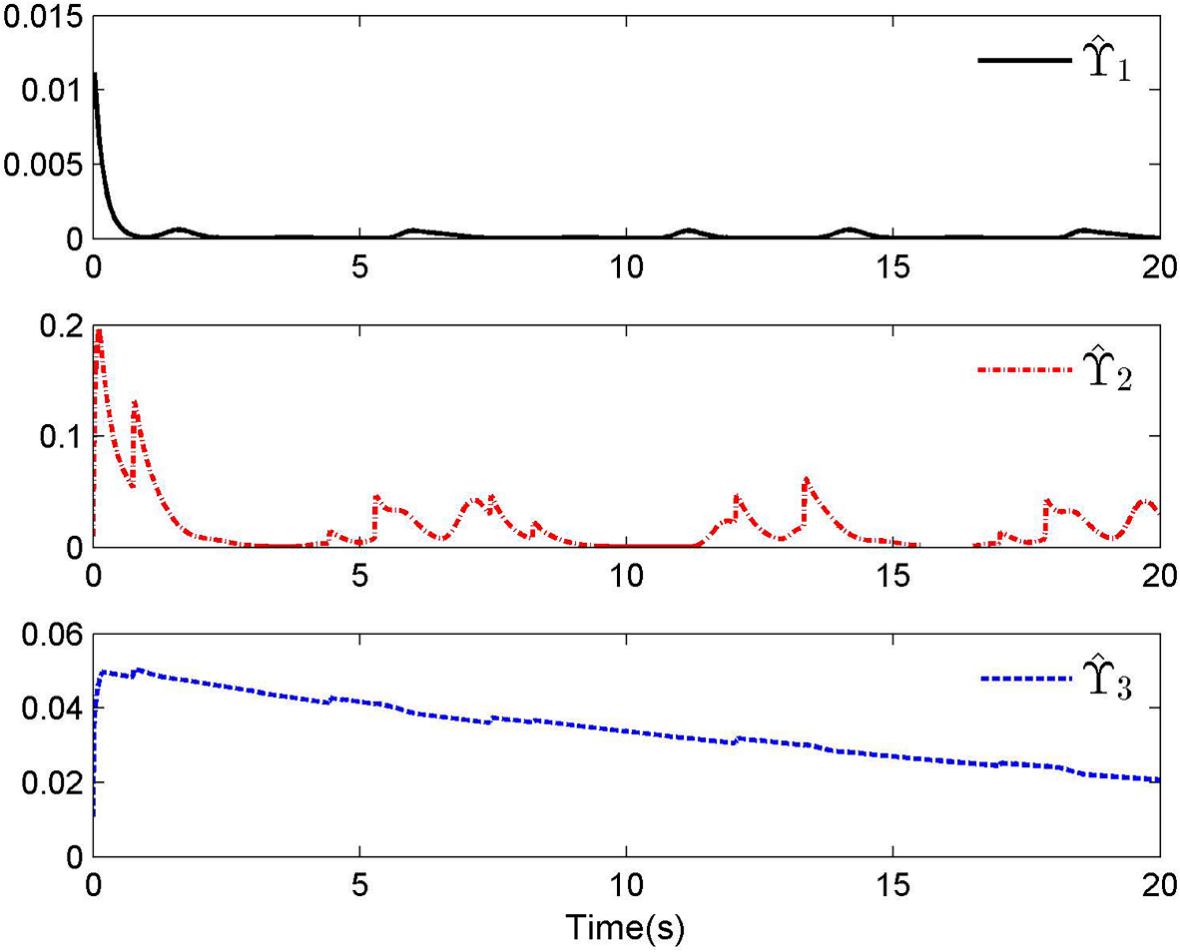
Adaptive control laws $\hat{\Upsilon }_{1}$, $\hat{\Upsilon }_{2}$ and $\hat{\Upsilon }_{3}$ for Case 2.

**Fig. 8:**
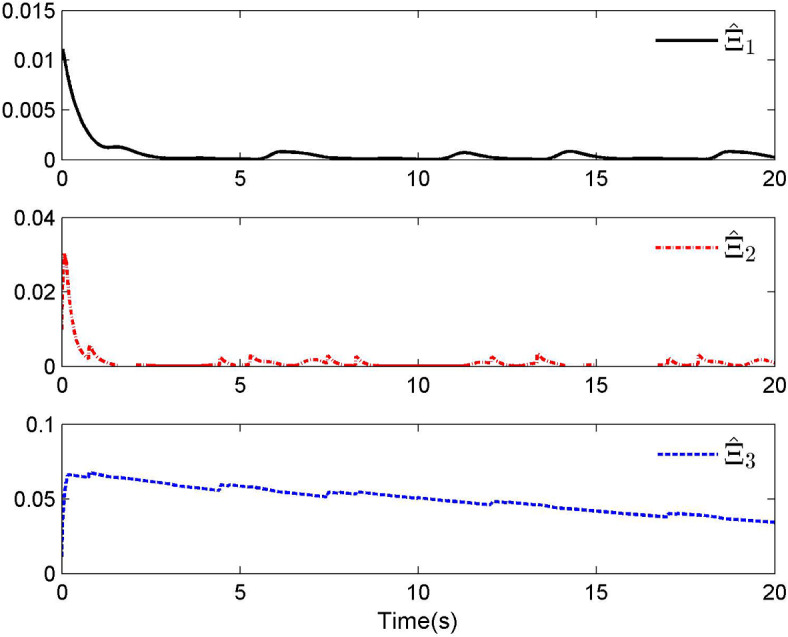
Adaptive control laws $\hat{\Xi }_{1}$, $\hat{\Xi }_{2}$ and $\hat{\Xi }_{3}$ for Case 2.

76and $d_{0} (u)$ is bounded. In this case, let $\delta_{l} = 1.2$, $\delta_{r} = 2.5$, $\text{Q}_{l} = 0.5$ and $\text{Q}_{r} = 1.0$. The simulation results are given in Figs. 5, 6, 7 and 8.

Case 3

${\mathcal{D}}(u)$ is selected as a type of unknown quantization input^32^, which is described as

77 $${\mathcal{D}}(u) = \left\{ {\begin{array}{*{20}c} {u_{m} {\text{sign}}(u(t))} & {\frac{{u_{m} }}{1 + \iota } < \left| {u(t)} \right| \le u_{m} ,\;\dot{u}(t) < 0\;{\text{or}}\;u_{m} < \left| {u(t)} \right| \le \frac{{u_{m} }}{1 - \iota },\;\dot{u}(t) > 0} \\ {u_{m} (1 + \iota ){\text{sign}}(u(t))} & {u_{m} < \left| {u(t)} \right| \le \frac{{u_{m} }}{1 - \iota },\;\dot{u}(t) < 0\;{\text{or}}\;\frac{{u_{m} }}{1 - \iota } < \left| {u(t)} \right| \le \frac{{u_{m} (1 + \iota )}}{1 - \iota },\;\dot{u}(t) > 0} \\ 0 & {0 \le \left| {u(t)} \right| < \frac{{u_{0} }}{1 + \iota },\;\dot{u}(t) < 0\;{\text{or}}\;\frac{{u_{0} }}{1 + \iota } \le \left| {u(t)} \right| \le u_{0} ,\;\dot{u}(t) > 0} \\ {{\mathcal{D}}(u(t^{ - } ))} & {{\text{otherwise}}} \\ \end{array} } \right.$$

where $u_{m} = \omega^{(1 - m)} u_{0}$, $m = 1,2, \ldots$, $\omega \in (0,1)$ and $\iota = {{(1 - \omega )} \mathord{\left/ {\vphantom {{(1 - \omega )} {(1 + \omega )}}} \right. \kern-0pt} {(1 + \omega )}}$ with $\omega$ being called as the quantization density and $u_{0} > 0$ being a design parameter.

**Fig. 9:**
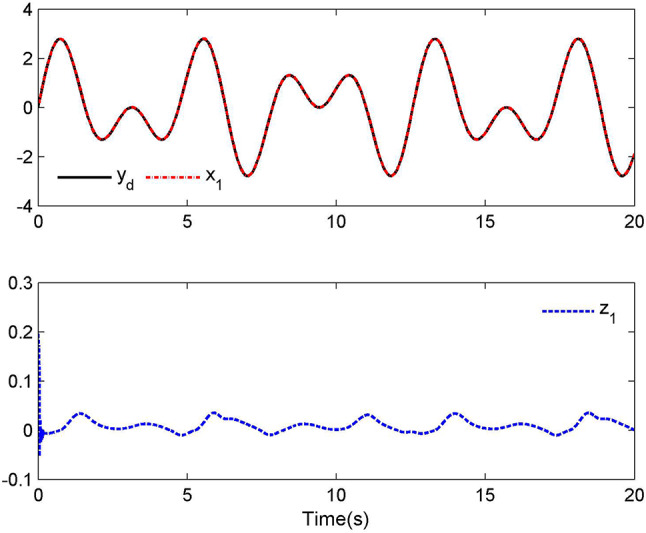
Tracking performance for Case 3.

**Fig. 10:**
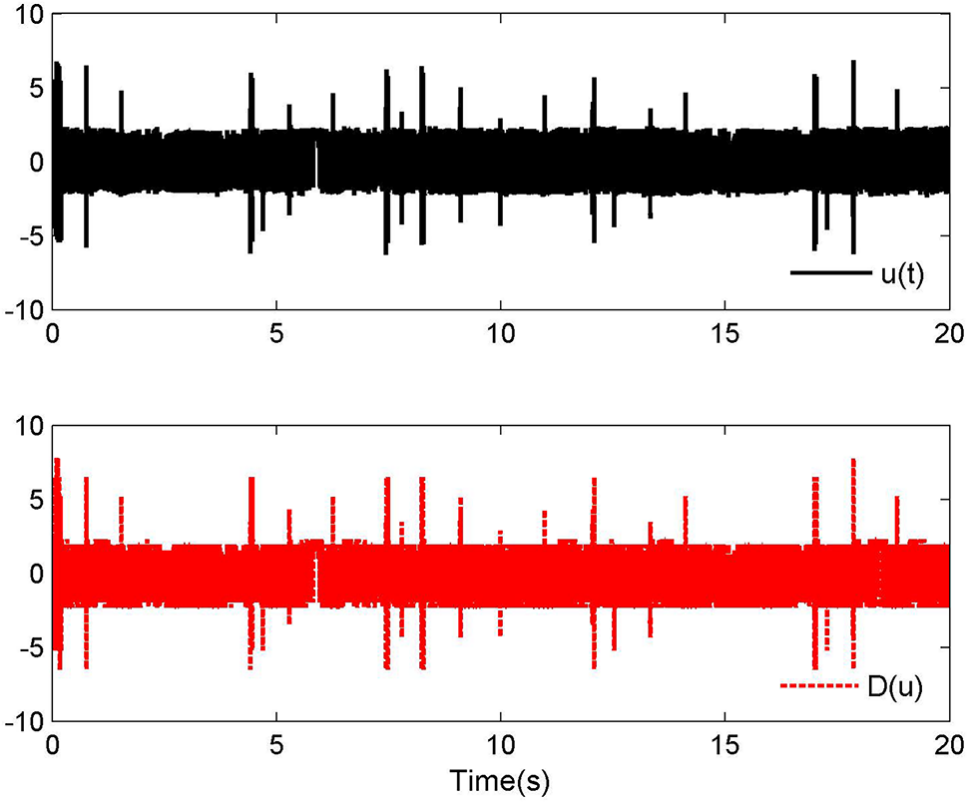
Control laws $u(t)$ and ${\mathcal{D}}(u)$ for Case 3.

**Fig. 11:**
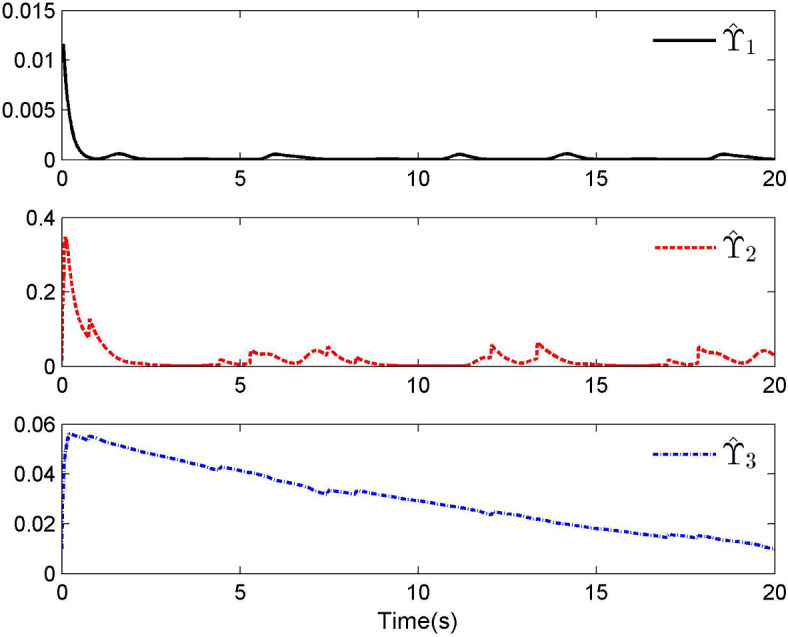
Adaptive control laws $\hat{\Upsilon }_{1}$, $\hat{\Upsilon }_{2}$ and $\hat{\Upsilon }_{3}$ for Case 3.

**Fig. 12:**
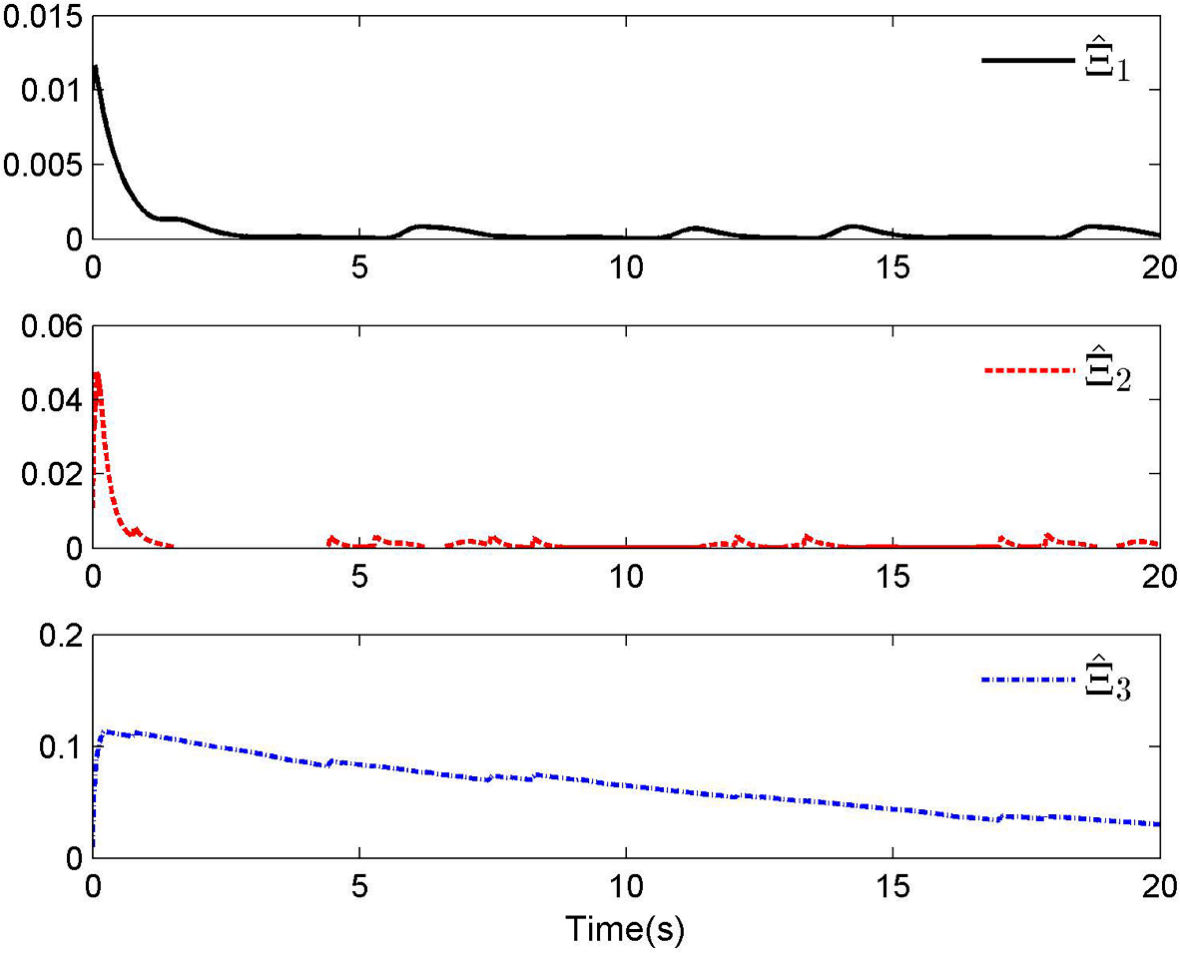
Adaptive control laws $\hat{\Xi }_{1}$, $\hat{\Xi }_{2}$ and $\hat{\Xi }_{3}$ for Case 3.

Compared with (2), we have $\kappa_{1} (t) = {\rm N}(u)$ and $\kappa_{2} (t) = {\rm H}(t)$, where ${\rm N}(u)$ and ${\rm H}(t)$ satisfy that $0 < 1 - \iota \le {\rm N}(u) \le 1 + \iota$ and $\left| {{\rm H}(t)} \right| \le u_{0}$. In this case, let $u_{0} = 0.05$, $m = 100$, $\omega = 0.2$ and $\iota = {2 \mathord{\left/ {\vphantom {2 3}} \right. \kern-0pt} 3}$. The simulation results are displayed in Figs. 9, 10, 11 and 12.

By applying the designed control law, tracking performance curves under three different input nonlinearities are shown in Figs. 1, 5, and 9, respectively. It is evident that the system’s output can effectively follow the specified desired trajectory. While the initial tracking error is comparatively significant at the onset of the simulation, it is observed that, as the simulation advances, the tracking error tends to converge to a small neighborhood of zero within a fixed time. Control laws $u(t)$ and ${\mathcal{D}}(u)$ under three different input nonlinearities are shown in Fig. 2, 6, and 10, respectively. Although these control signals are not smooth, they are all bounded. As stated in Remark 2, we tend to focus more on tracking performance in the selection of control performance and control signals. Additionally, under three different input nonlinearities, the curves of adaptive control laws $\hat{\Upsilon }_{1}$, $\hat{\Upsilon }_{2}$ and $\hat{\Upsilon }_{3}$ are depicted in Figs. 3, 7 and 11, and $\hat{\Xi }_{1}$, $\hat{\Xi }_{2}$ and $\hat{\Xi }_{3}$ are displayed in Figs. 4, 8 and 12, respectively. Obviously, these adaptive control laws are bounded.

The simulation results clearly indicate that all signals in the closed-loop system remain bounded, and the tracking error can converge to a small neighborhood of zero within a fixed time. The efficacy of the control law presented in this paper is thoroughly substantiated.

## Conclusion

In this paper, The FTTC issue of uncertain HONSs with time-varying parameters and unknown input nonlinearity is addressed. The NN approximation method is used to deal with unknown nonlinear dynamics, and the adaptive parameter estimation approach is considered to estimate unknown parameters. Meantime, the NGF technique is utilized to handle the unknown control gain caused by unknown input nonlinearity. Furthermore, an adaptive NN-based FTTC strategy is proposed under the backstepping control framework. By introducing three different types of input nonlinearity, the simulation results can effectively validate the efficacy of the developed control method.

This paper considered the uncertain HONSs and the FTTC problem is achieved by combining the NN approximation technique and the NGF technique. However, this paper neither considers the case of HONSs with unknown time-varying coefficients nor explores the issue of prescribed performance. This will be our future work.

## Data Availability

The data is available from the corresponding author on reasonable request.
